# Supplementary material for: Prediction of nonlayered oxide monolayers as flexible high-κ dielectrics with negative Poisson’s ratios
Source: Nat Commun. 2023 Oct 17;14:6555. doi: 10.1038/s41467-023-42312-4 (PMC10582060; doi:10.1038/s41467-023-42312-4)
Supplement: Supplementary file 1 — Supplementary Information [file 41467_2023_42312_MOESM1_ESM.pdf]

Supplementary Information

Prediction of nonlayered oxide monolayers as flexible high-κ dielectrics with negative Poisson's ratios

Yue Hu[1,*], Jingwen Jiang[2], Peng Zhang[1,3], Zhuang Ma[1], Fuxin Guan[4], Da Li[1], Zhengfang Qian[1,3], Xiuwen Zhang[1,3,5,*] and Pu Huang[1,3,*]

[1] *Key Laboratory of Optoelectronic Devices and Systems of Ministry of Education and Guangdong Province, College of Physics and Optoelectronic Engineering, Shenzhen University, 518060, Shenzhen, China*
[2] *School of Information Engineering, Jiangmen Polytechnic, Jiangmen, China*
[3] *State Key Laboratory of Radio Frequency Heterogeneous Integration, Shenzhen University, 518060 Shenzhen, China*
[4] *Department of Physics, University of Hong Kong, Hong Kong, China*

* Corresponding author: *yuehuphd@szu.edu.cn, xiuwenzhang@szu.edu.cn, arvin_huang@szu.edu.cn*

[5] *Current address: Renewable and Sustainable Energy Institute, University of Colorado, Boulder, Colorado 80309, USA*

Supplementary Notes

Workflow. We start from 1,921 binary metal oxides in Materials Project[1] and select the experimentally stable nonlayered compounds to build the input dataset. From the generated dataset, we remove the materials with more than 10 atoms in the unit cell and the compounds containing *f*-valence electrons (in consideration of the computational efficiency and the possible computational difficulty due to *f*-valence electrons). We then add the oxide dielectrics TiO_2, ZrO_2 and HfO_2 to the data list. Finally, we arrive at a mixed set of 48 nonlayered bulk oxides, including a sizable portion of transition metal, post–transition metal, alkali-metal and alkaline-earth metal oxides, among which there are 9 magnetic oxides, see Supplementary Table 2. At this point, we have done the preparatory work. The workflow diagram for this work is summarized in Supplementary Figure 1.

Nonlayered Oxides Exfoliation. For the extraction of 2D materials from non-layered bulks, the first step is to determine the promising exfoliated crystal planes. The strategy is to identify the crystal planes with large difference between in-plane and interplanar interactions. A primary tool is the set of interplanar spacing (d_{hkl}) and packing ratio of different planes. We select the close-packed planes and nearly close-packed planes with large d_{hkl}, indicating that there may be a relatively weak out-of-plane interaction and strong in-pane bonding. In the second step, we rotate the selected crystal plane (*h k l*) to the (0 0 1) plane, in preparation for the subsequent exfoliation calculations. In the last step, we extract 2D material from its bulk precursor by gradually increasing the tensile stress on the selected crystal plane. The specific procedure is as follows: we stretch the crystal along the *z*-direction in steps of 5% strain, during which the in-plane lattice constants *a*, *b* and all the atoms in the system are fully optimized. In addition, to complete the extraction of 2D material from its 3D

precursor, it usually takes 22 to 130 stretching steps, for all the exfoliation cases we considered.

Supplementary Discussion

Mechanistic Explanation. Negative Poisson's ratio (NPR) effect of GeO_2 (101). When the GeO_2 monolayer is stretched in the *x*-direction, the angle θ_1 increases, together with the lattice constant in the *y*-direction decreases, see Supplementary Figure 36(a). The contraction of lattice in the *y*-direction results in a decrease in the angle θ_2, see Supplementary Figure 36 (b), the monolayer thickness in the *z*-direction thus increases, and vice versa, leading to NPR effect. Red and grey spheres (sticks) denote O and the Ge atoms, respectively.

NPR effect for MO_2 (110) (M=Os, Pb, Pd, and Rh). When the MO_2 (110) monolayers are stretched in the *y*-direction, the angle θ_3 decreases and the lattice in the *x*-direction contracts, see Supplementary Figure 37(a). The contraction of lattice in the *x*-direction leads to the out-of-plane angle θ_4 decreases and the monolayer expands in the *z*-direction (see Supplementary Figure 37(b)), and vice versa, leading to NPR effect. Red and grey spheres (sticks) denote O and metal atoms, respectively.

Biased-Poisson's ratio effect of WO_3 (110). When WO_3 (110) monolayer is compressed in the *x* direction, the angle θ_5 in the *x z* plane decreases, making O_1 atom move down along the *z* axis, the WO_3 (110) monolayer thus expands in the *z*-direction, see Supplementary Figure 38(a). In turn, if WO_3 (110) monolayer is stretched in the *x*-direction, WO_3 (110) still expands in the *z*-direction. This is because the lattice expansion in the *x*-direction will cause the lattice contraction in the *y*-direction, see Supplementary Figure 38(b), which makes the angle θ_6 in the *yz* plane decrease and O_2 atom move down along the *z*-direction, WO_3 (110) monolayer thus still expands in the *z*-direction. The mechanical response in the *z*-direction to the uniaxial strain in the *x* and *y* directions are fully identical due to the structural isotropy in the *x* and *y* directions.

Biased-Poisson's ratio effect of AgO/CuO (101). when the AgO/CuO (101) monolayers are compressed in the *x*-direction, the angle θ_7 decreases, see the left panel in Supplementary Figure 39(a), which leads to the monolayer expands in the *z*-direction; while if the MO (101) monolayers are stretched in the *x*-direction, the lattice in the *y*-direction contracts, see Supplementary Figure 39(b), and the angle θ_8 decreases, which also causes the MO (101) monolayers to expand in the *z*-direction.

Supplementary Figures

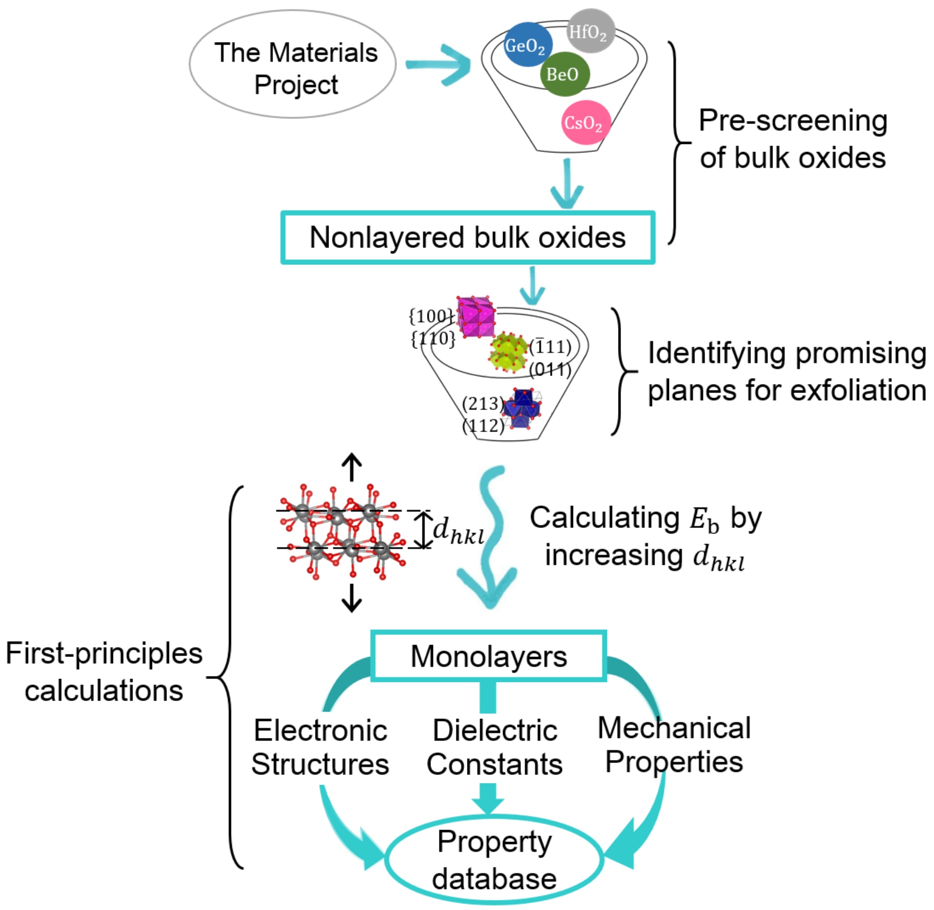

Supplementary Figure 1. **Workflow diagram.** The experimentally stable nonlayered bulk oxides collected from the Materials Project are filtered for the ensuing exfoliation and physical properties calculations.

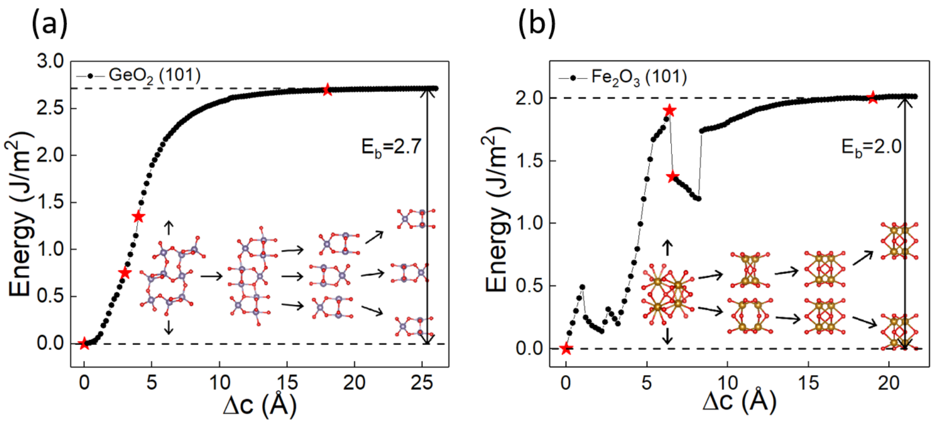

Supplementary Figure 2. **Energy versus the change in the lattice constant *c* for (a) GeO_2 (101) and (b) Fe_2O_3 (101).** For clarity, the energy and lattice constant *c* at zero strain is set to zero. Inset: The crystal structures correspond to the red five-stars on the energy curve from left to right.

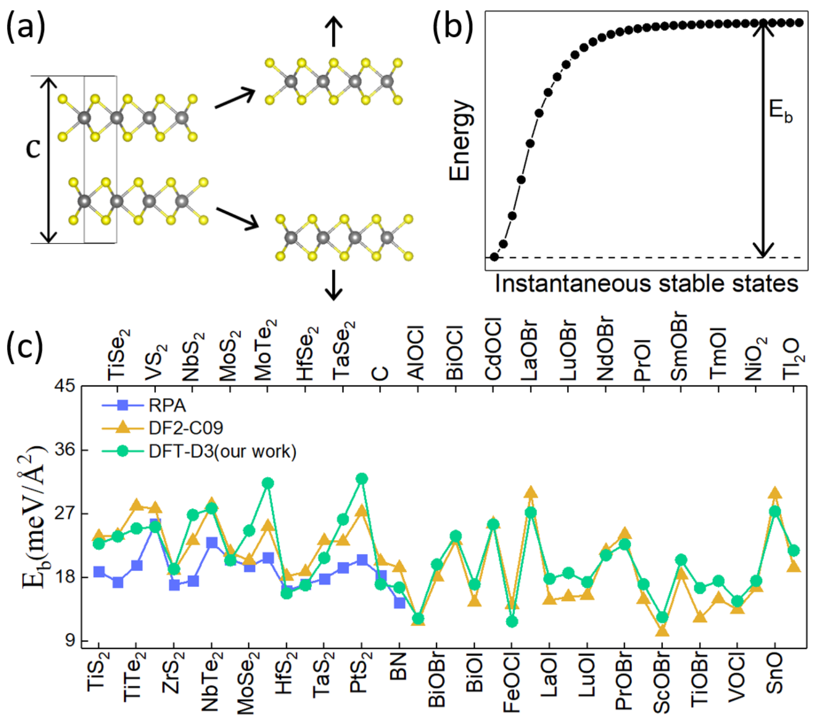

Supplementary Figure 3. Binding energy for layered materials. (a) Procedure for calculating the interlayer binding energy (E_b) of layered materials by increasing the lattice constant *c*. (b) Schematic illustration of a binding energy curve. (c) Comparison chart for E_b of 38 layered materials calculated by DF2-C09[6] and DF2-D3 (our work) functionals, 17 of which are also calculated by the random phase approximation (RPA)[7] functional.

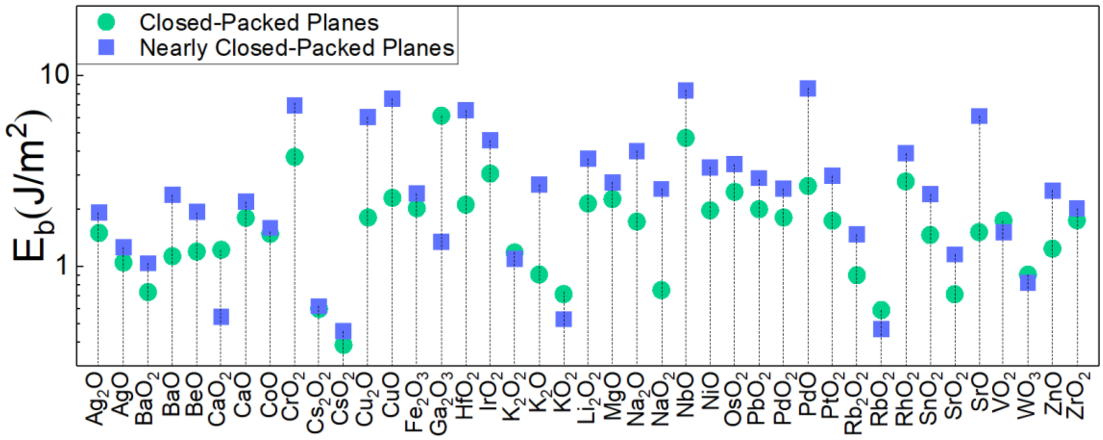

Supplementary Figure 4. Comparison chart for the interplanar binding energy (E_b) of close-packed and nearly close-packed planes. It is easily seen that the E_b of close-packed plane is in general lower than that of the nearly close-packed plane.

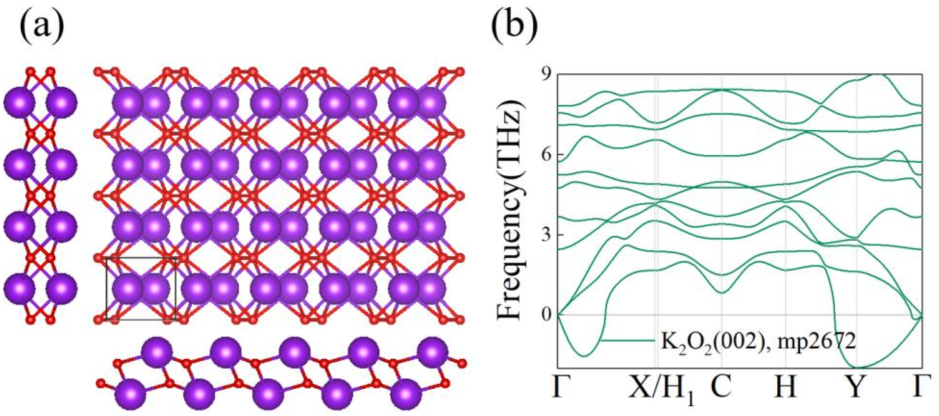

Supplementary Figure 5. Crystal structure and phonon dispersion. (a) Orthographic projections: views of K_2O_2 (002) as seen from the x axis (left), the y axis (bottom) and the z axis (center). The primitive cell is marked by solid box. (b) Phonon frequencies of K_2O_2 (002) along a high-symmetry path.

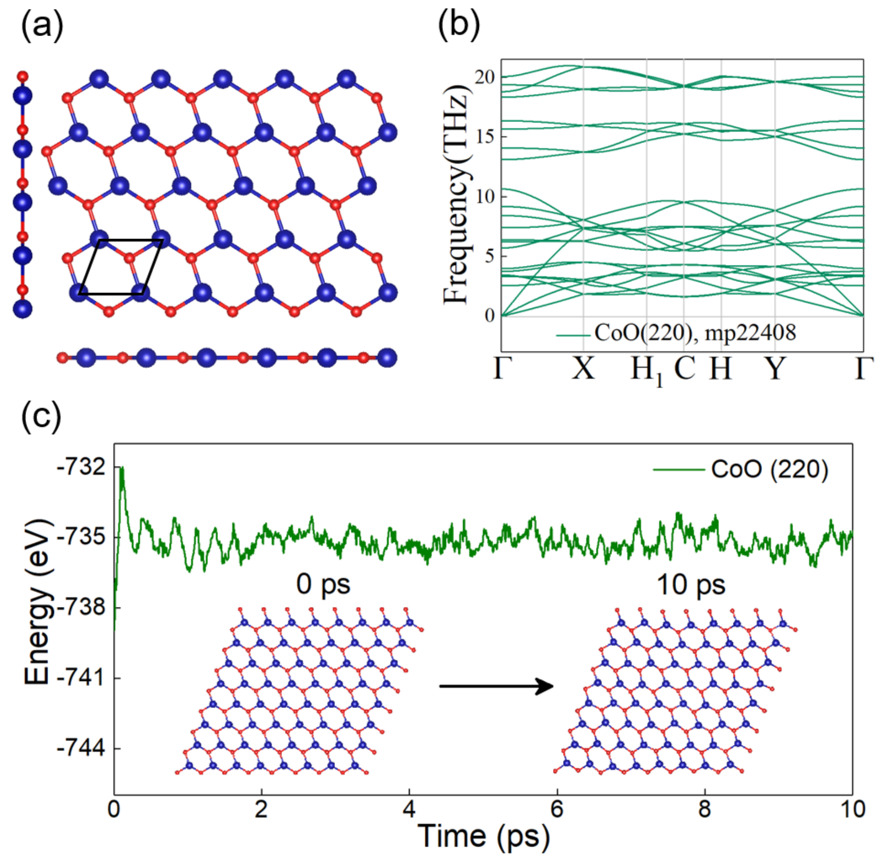

Supplementary Figure 6. Crystal structure, phonon dispersion and AIMD simulation. (a) Orthographic projections: views of CoO (220) as seen from the x axis (left), the y axis (bottom) and the z axis (center). The primitive cell is marked by solid box. (b) Phonon dispersion: phonon frequencies of CoO (220) along a high-symmetry path. (c) Total system energy fluctuation with simulation time, where insets are crystal structures at 0 ps and 10 ps, respectively.

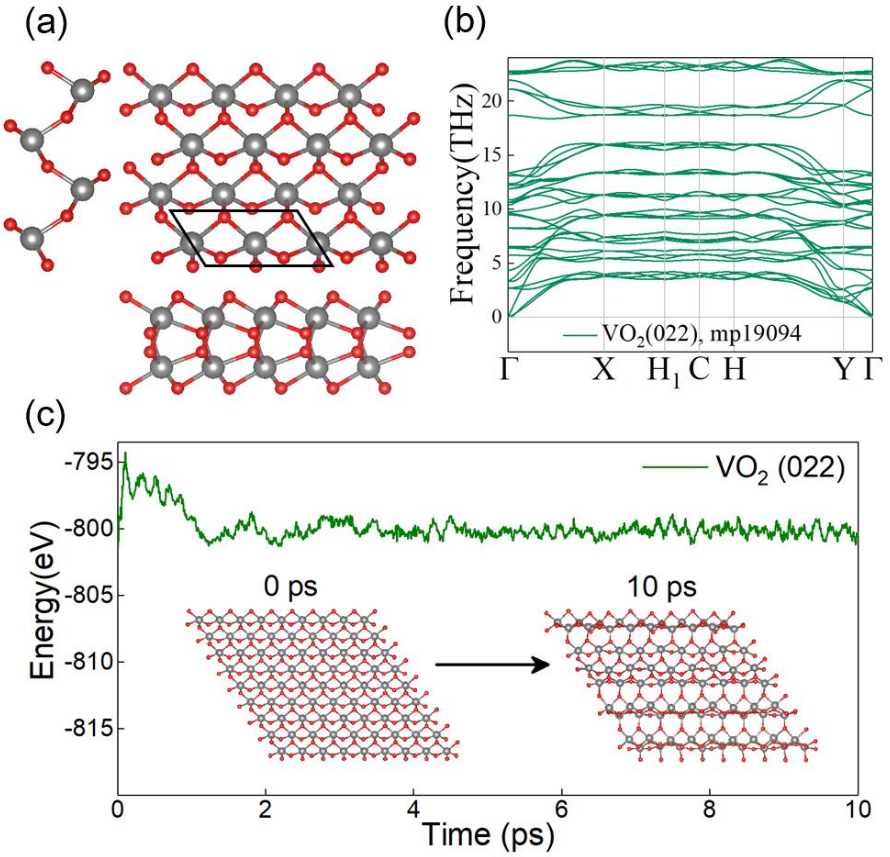

Supplementary Figure 7. Crystal structure, phonon dispersion and AIMD simulation. (a) Orthographic projections: views of VO_2 (022) as seen from the x axis (left), the y axis (bottom) and the z axis (center). The primitive cell is marked by solid box. (b) Phonon dispersion: phonon frequencies of VO_2 (022) along a high-symmetry path. (c) Total system energy fluctuation with simulation time, where insets are crystal structures at 0 ps and 10 ps, respectively.

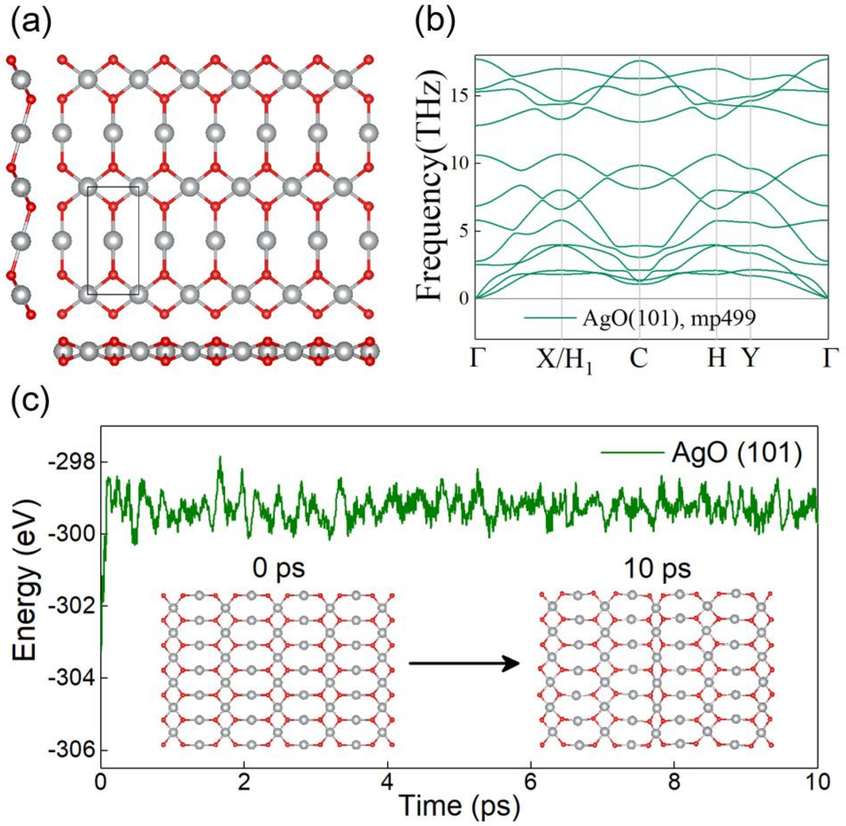

Supplementary Figure 8. Crystal structure, phonon dispersion and AIMD simulation. (a) Orthographic projections: views of AgO (101) as seen from the x axis (left), the y axis (bottom) and the z axis (center). The primitive cell is marked by solid box. (b) Phonon dispersion: phonon frequencies of AgO (101) along a high-symmetry path. (c) Total system energy fluctuation with simulation time, where insets are crystal structures at 0 ps and 10 ps, respectively.

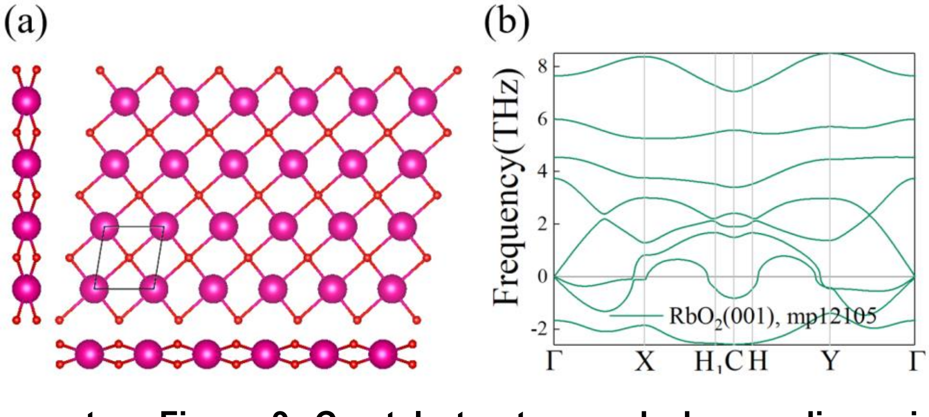

Supplementary Figure 9. Crystal structure and phonon dispersion. (a) Orthographic projections: views of RbO_2 (001) as seen from the x axis (left), the y axis (bottom) and the z axis (center). The primitive cell is marked by solid box. (b) Phonon dispersion: phonon frequencies of RbO_2 (001) along a high-symmetry path.

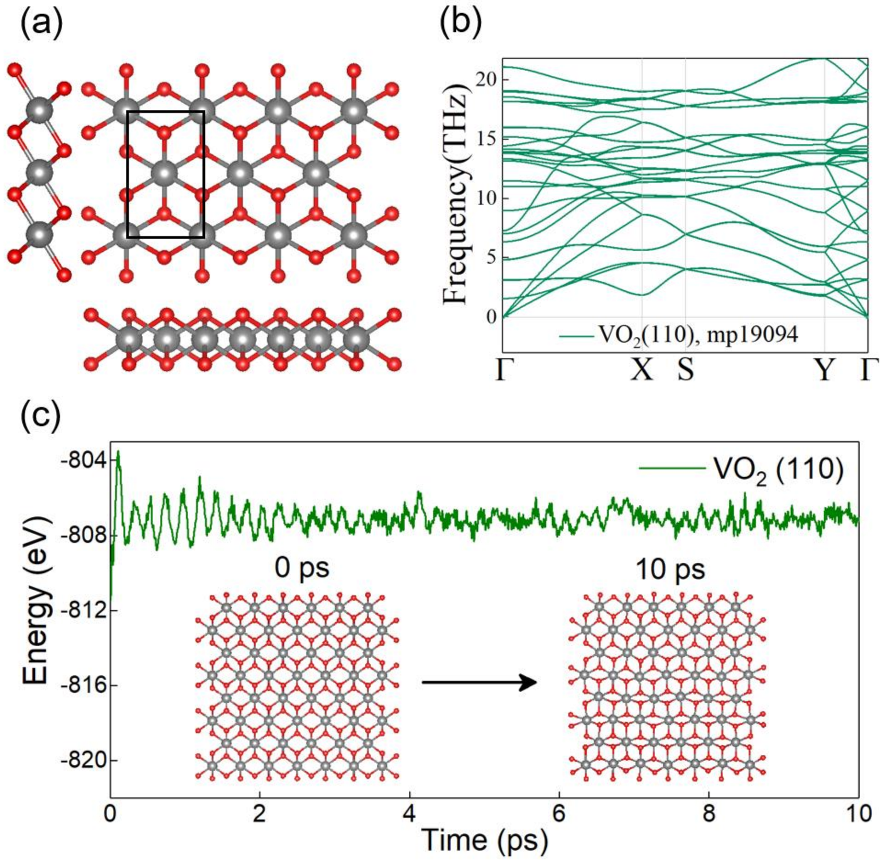

Supplementary Figure 10. **Crystal structure, phonon dispersion and AIMD simulation.** (a) Orthographic projections: views of VO_2 (110) as seen from the x axis (left), the y axis (bottom) and the z axis (center). The primitive cell is marked by solid box. (b) Phonon dispersion: phonon frequencies of VO_2 (110) along a high-symmetry path. (c) Total system energy fluctuation with simulation time, where insets are crystal structures at 0 ps and 10 ps, respectively.

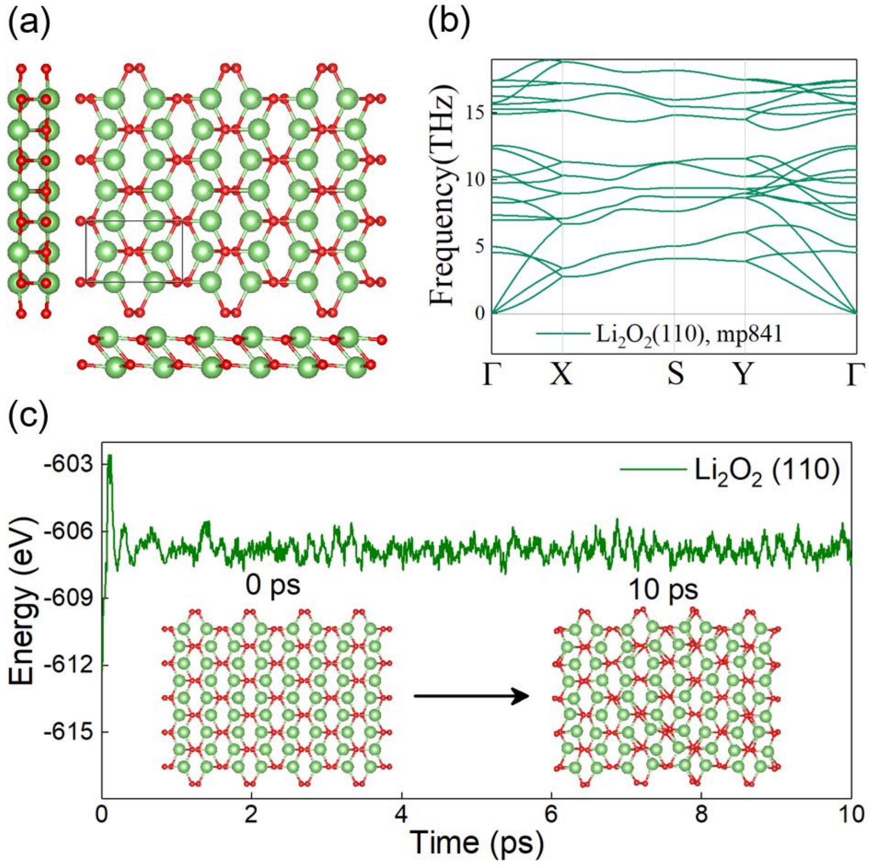

Supplementary Figure 11. Crystal structure, phonon dispersion and AIMD simulation. (a) Orthographic projections: views of Li_2O_2 (110) as seen from the x axis (left), the y axis (bottom) and the z axis (center). The primitive cell is marked by solid box. (b) Phonon dispersion: phonon frequencies of Li_2O_2 (110) along a high-symmetry path. (c) Total system energy fluctuation with simulation time, where insets are crystal structures at 0 ps and 10 ps, respectively.

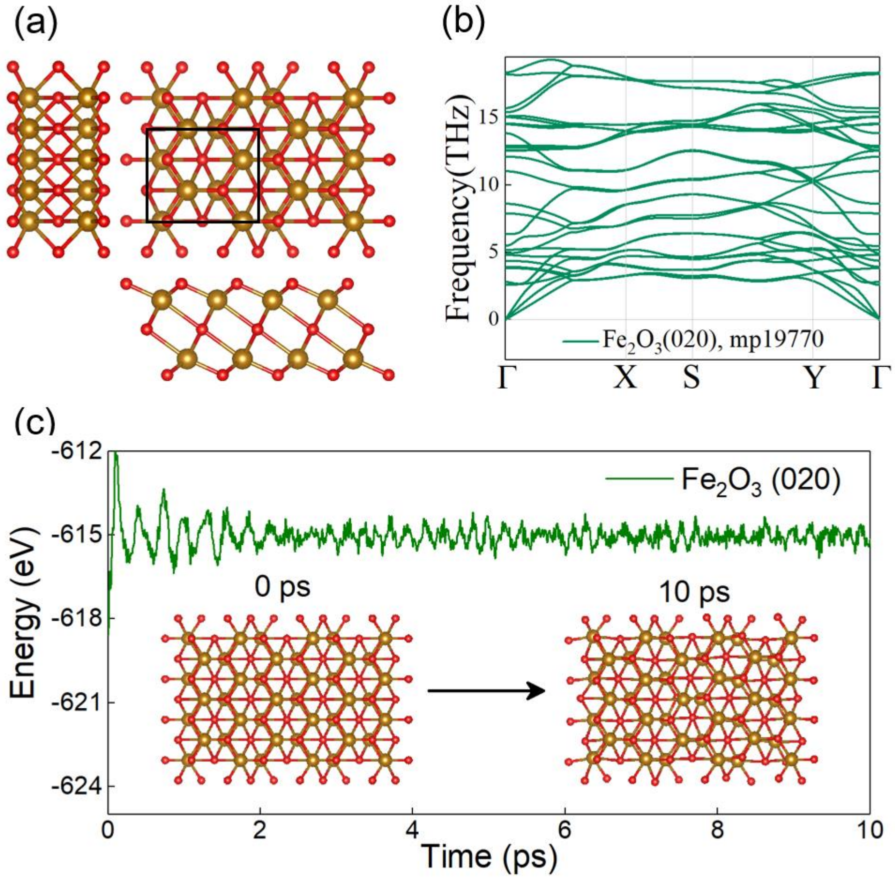

Supplementary Figure 12. Crystal structure, phonon dispersion and AIMD simulation. (a) Orthographic projections: views of Fe_2O_3 (020) as seen from the x axis (left), the y axis (bottom) and the z axis (center). The primitive cell is marked by solid box. (b) Phonon dispersion: phonon frequencies of Fe_2O_3 (020) along a high-symmetry path. (c) Total system energy fluctuation with simulation time, where insets are crystal structures at 0 ps and 10 ps, respectively.

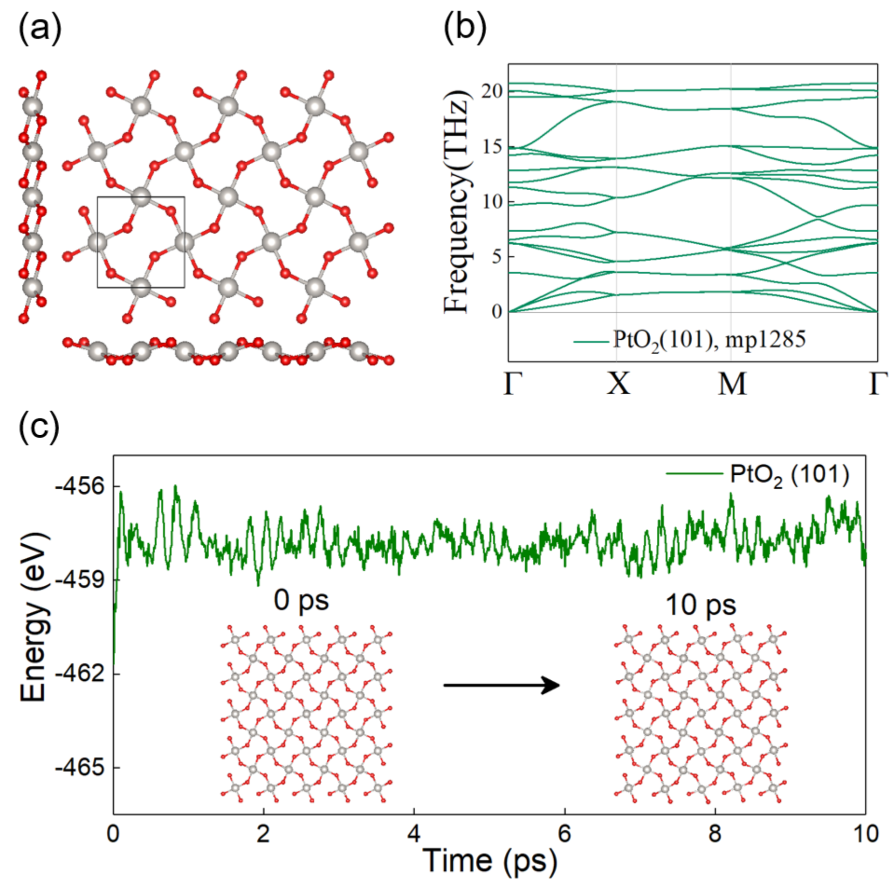

Supplementary Figure 13. Crystal structure, phonon dispersion and AIMD simulation. (a) Orthographic projections: views of PtO_2 (101) as seen from the x axis (left), the y axis (bottom) and the z axis (center). The primitive cell is marked by solid box. (b) Phonon dispersion: phonon frequencies of PtO_2 (101) along a high-symmetry path. (c) Total system energy fluctuation with simulation time, where insets are crystal structures at 0 ps and 10 ps, respectively.

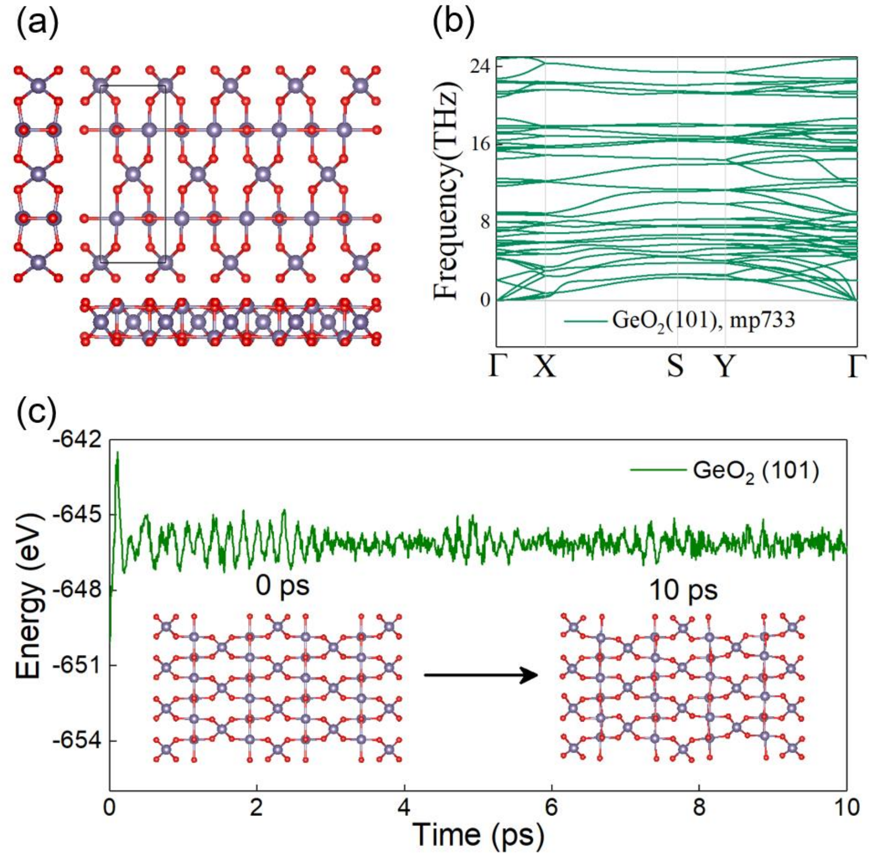

Supplementary Figure 14. Crystal structure, phonon dispersion and AIMD simulation. (a) Orthographic projections: views of GeO_2 (101) as seen from the x axis (left), the y axis (bottom) and the z axis (center). The primitive cell is marked by solid box. (b) Phonon dispersion: phonon frequencies of GeO_2 (101) along a high-symmetry path. (c) Total system energy fluctuation with simulation time, where insets are crystal structures at 0 ps and 10 ps, respectively.

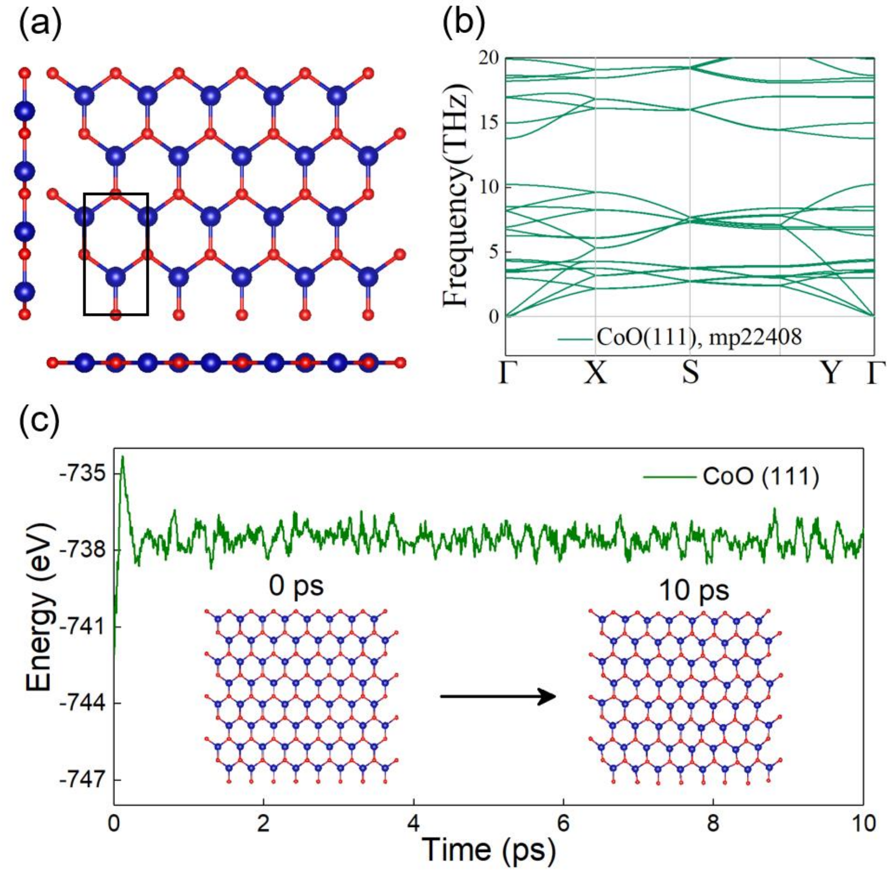

Supplementary Figure 15. **Crystal structure, phonon dispersion and AIMD simulation.** (a) Orthographic projections: views of CoO (111) as seen from the x axis (left), the y axis (bottom) and the z axis (center). The primitive cell is marked by solid box. (b) Phonon dispersion: phonon frequencies of CoO (111) along a high-symmetry path. (c) Total system energy fluctuation with simulation time, where insets are crystal structures at 0 ps and 10 ps, respectively.

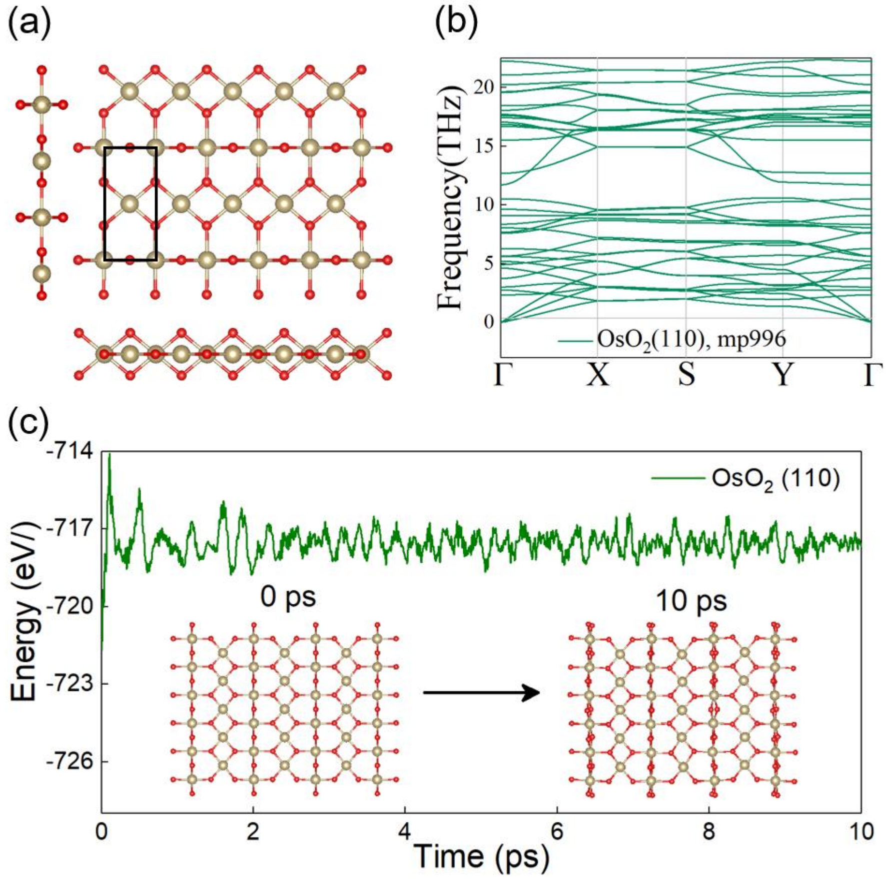

Supplementary Figure 16. Crystal structure, phonon dispersion and AIMD simulation. (a) Orthographic projections: views of OsO_2 (110) as seen from the x axis (left), the y axis (bottom) and the z axis (center). The primitive cell is marked by solid box. (b) Phonon dispersion: phonon frequencies of OsO_2 (110) along a high-symmetry path. (c) Total system energy fluctuation with simulation time, where insets are crystal structures at 0 ps and 10 ps, respectively.

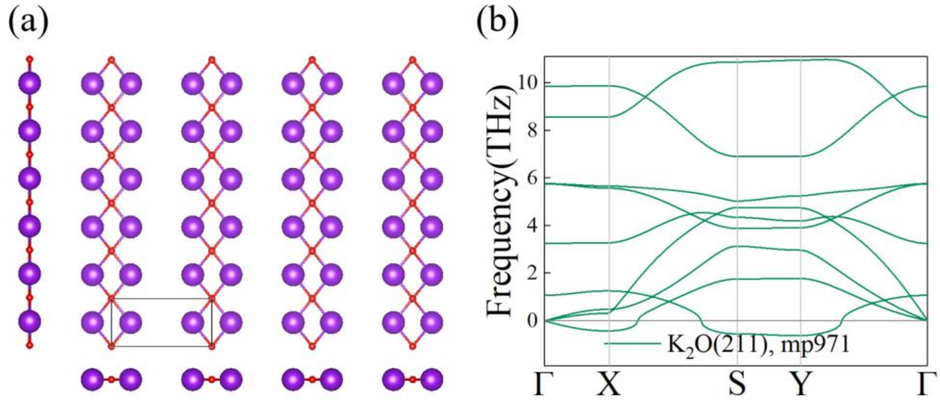

Supplementary Figure 17. Crystal structure and phonon dispersion. (a) Orthographic projections: views of K_2O (211) as seen from the x axis (left), the y axis (bottom) and the z axis (center). The primitive cell is marked by solid box. (b) Phonon dispersion: phonon frequencies of K_2O (211) along a high-symmetry path.

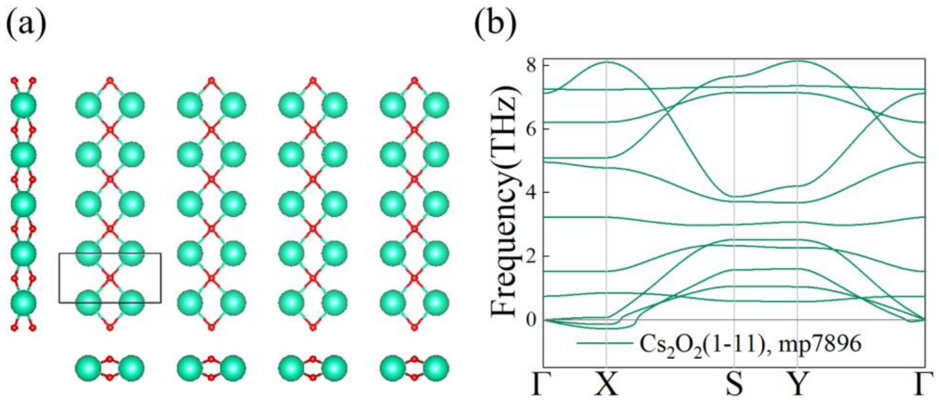

Supplementary Figure 18. Crystal structure and phonon dispersion. (a) Orthographic projections: views of Cs_2O_2 (1$\bar{1}$1) as seen from the x axis (left), the y axis (bottom) and the z axis (center). The primitive cell is marked by solid box. (b) Phonon dispersion: phonon frequencies of Cs_2O_2 (1$\bar{1}$1) along a high-symmetry path.

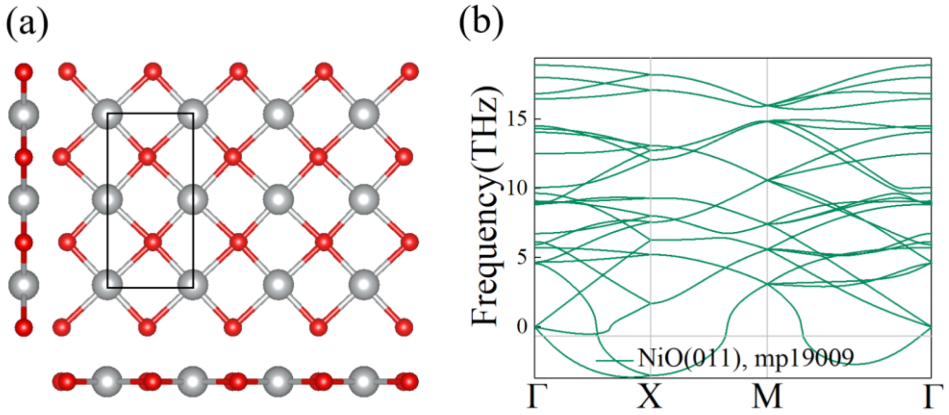

Supplementary Figure 19. **Crystal structure and phonon dispersion.** (a) Orthographic projections: views of NiO (011) as seen from the x axis (left), the y axis (bottom) and the z axis (center). The primitive cell is marked by solid box. (b) Phonon dispersion: phonon frequencies of NiO (011) along a high-symmetry path.

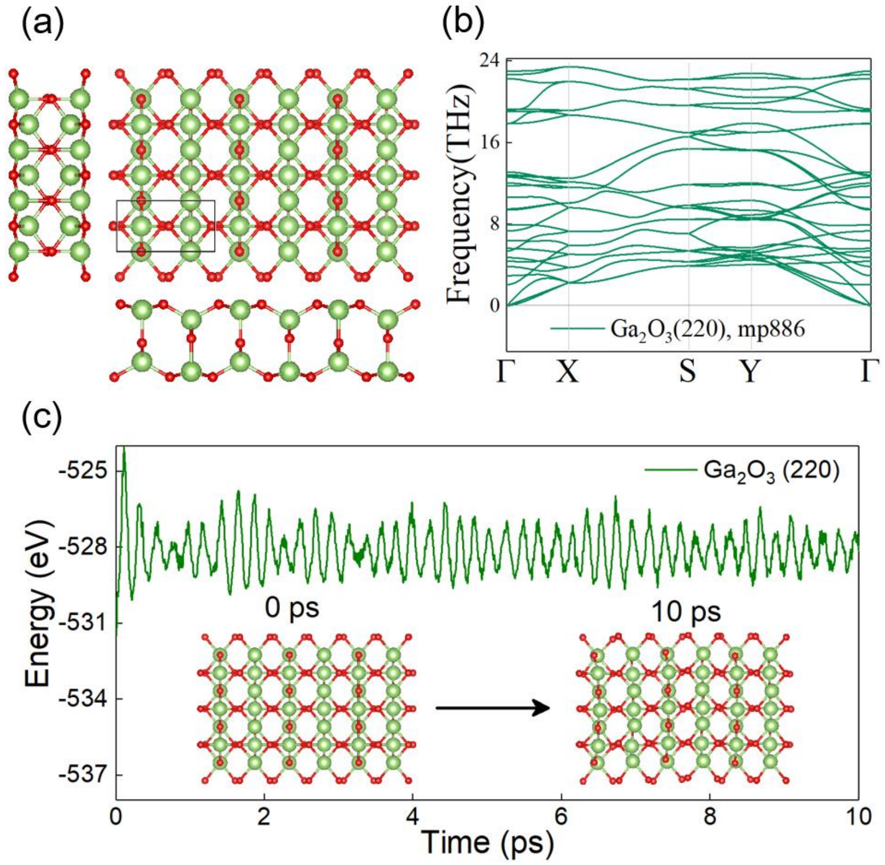

Supplementary Figure 20. Crystal structure, phonon dispersion and AIMD simulation. (a) Orthographic projections: views of Ga_2O_3 (220) as seen from the x axis (left), the y axis (bottom) and the z axis (center). The primitive cell is marked by solid box. (b) Phonon dispersion: phonon frequencies of Ga_2O_3 (220) along a high-symmetry path. (c) Total system energy fluctuation with simulation time, where insets are crystal structures at 0 ps and 10 ps, respectively.

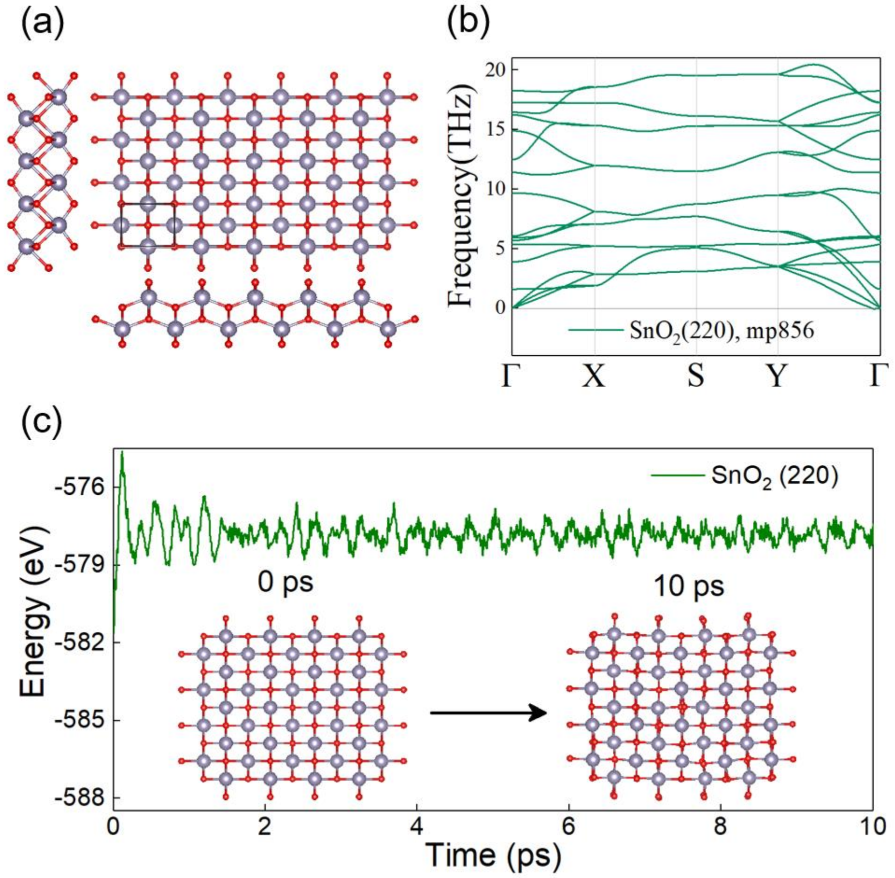

Supplementary Figure 21. Crystal structure, phonon dispersion and AIMD simulation. (a) Orthographic projections: views of SnO_2 (220) as seen from the x axis (left), the y axis (bottom) and the z axis (center). The primitive cell is marked by solid box. (b) Phonon dispersion: phonon frequencies of SnO_2 (220) along a high-symmetry path. (c) Total system energy fluctuation with simulation time, where insets are crystal structures at 0 ps and 10 ps, respectively.

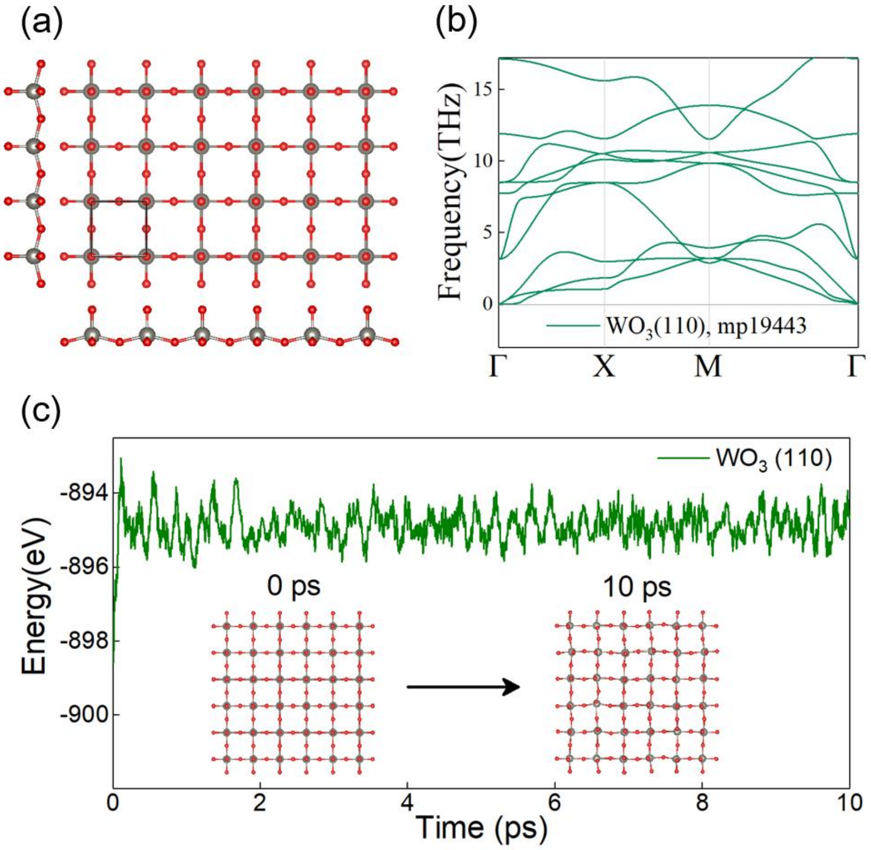

Supplementary Figure 22. Crystal structure, phonon dispersion and AIMD simulation. (a) Orthographic projections: views of WO_3 (110) as seen from the x axis (left), the y axis (bottom) and the z axis (center). The primitive cell is marked by solid box. (b) Phonon dispersion: phonon frequencies of WO_3 (110) along a high-symmetry path. (c) Total system energy fluctuation with simulation time, where insets are crystal structures at 0 ps and 10 ps, respectively.

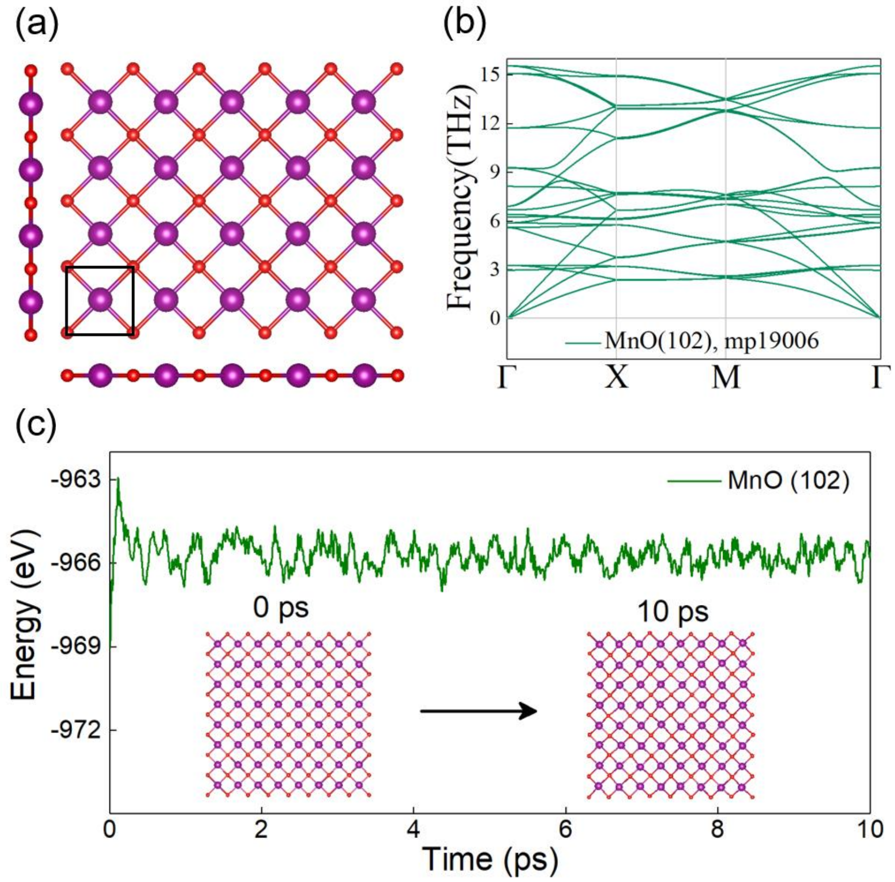

Supplementary Figure 23. Crystal structure, phonon dispersion and AIMD simulation. (a) Orthographic projections: views of MnO (102) as seen from the x axis (left), the y axis (bottom) and the z axis (center). The primitive cell is marked by solid box. (b) Phonon dispersion: phonon frequencies of MnO (102) along a high-symmetry path. (c) Total system energy fluctuation with simulation time, where insets are crystal structures at 0 ps and 10 ps, respectively.

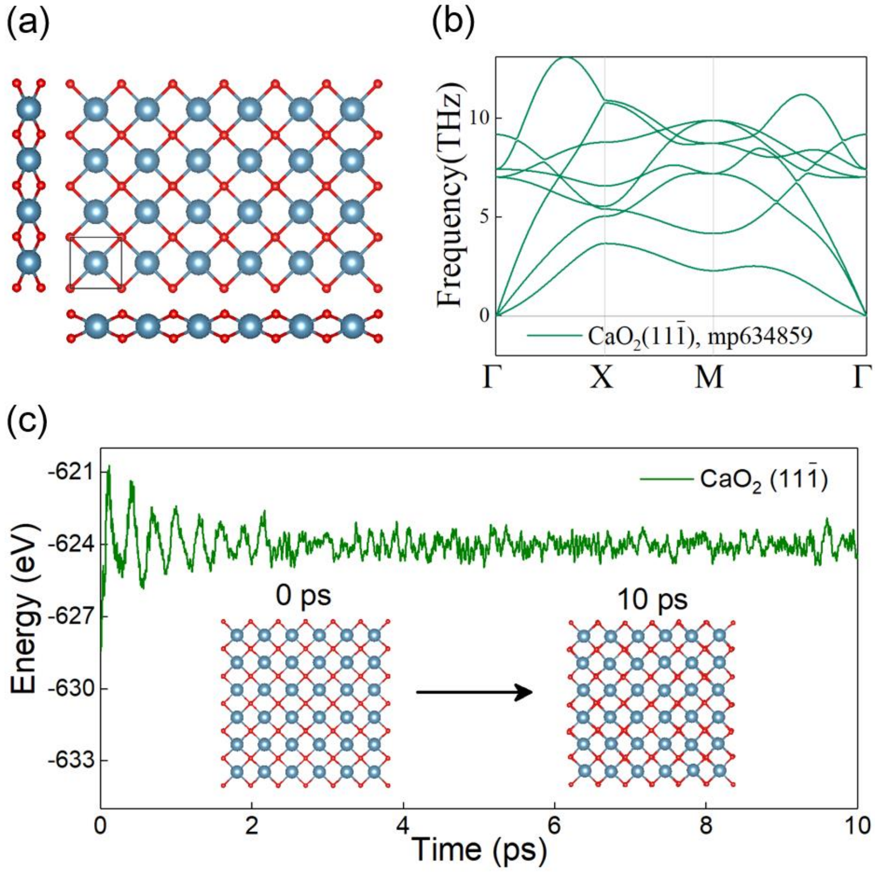

Supplementary Figure 24. Crystal structure, phonon dispersion and AIMD simulation. (a) Orthographic projections: views of CaO_2 $(11\bar{1})$ as seen from the x axis (left), the y axis (bottom) and the z axis (center). The primitive cell is marked by solid box. (b) Phonon dispersion: phonon frequencies of CaO_2 $(11\bar{1})$ along a high-symmetry path. (c) Total system energy fluctuation with simulation time, where insets are crystal structures at 0 ps and 10 ps, respectively.

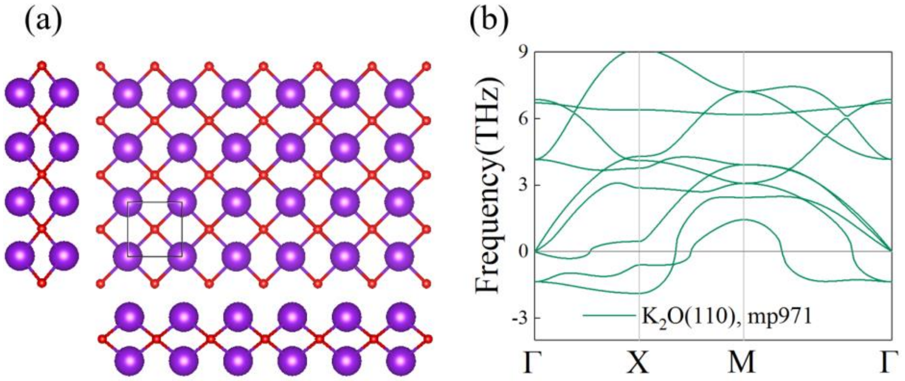

Supplementary Figure 25. Crystal structure and phonon dispersion. (a) Orthographic projections: views of K_2O (110) as seen from the x axis (left), the y axis (bottom) and the z axis (center). The primitive cell is marked by solid box. (b) Phonon dispersion: phonon frequencies of K_2O (110) along a high-symmetry path.

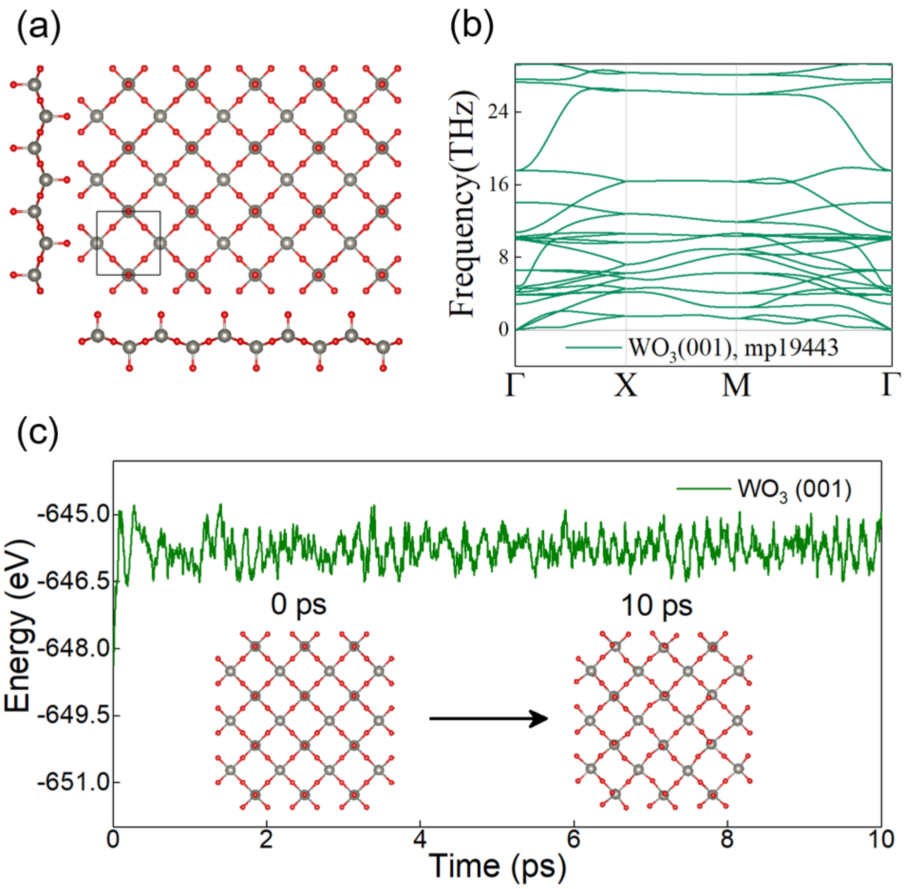

Supplementary Figure 26. Crystal structure, phonon dispersion and AIMD simulation. (a) Orthographic projections: views of WO_3 (001) as seen from the x axis (left), the y axis (bottom) and the z axis (center). The primitive cell is marked by solid box. (b) Phonon dispersion: phonon frequencies of WO_3 (001) along a high-symmetry path. (c) Total system energy fluctuation with simulation time, where insets are crystal structures at 0 ps and 10 ps, respectively.

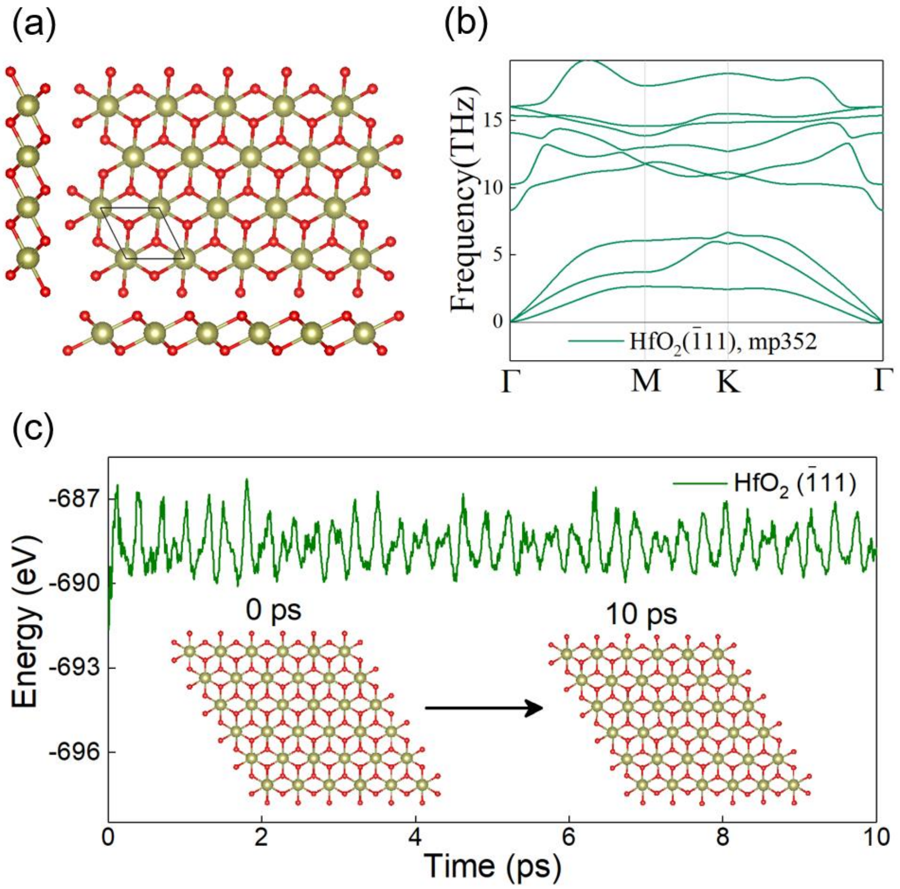

Supplementary Figure 27. Crystal structure, phonon dispersion and AIMD simulation. (a) Orthographic projections: views of HfO_2 ($\bar{1}11$) as seen from the x axis (left), the y axis (bottom) and the z axis (center). The primitive cell is marked by solid box. (b) Phonon dispersion: phonon frequencies of HfO_2 ($\bar{1}11$) along a high-symmetry path. (c) Total system energy fluctuation with simulation time, where insets are crystal structures at 0 ps and 10 ps, respectively.

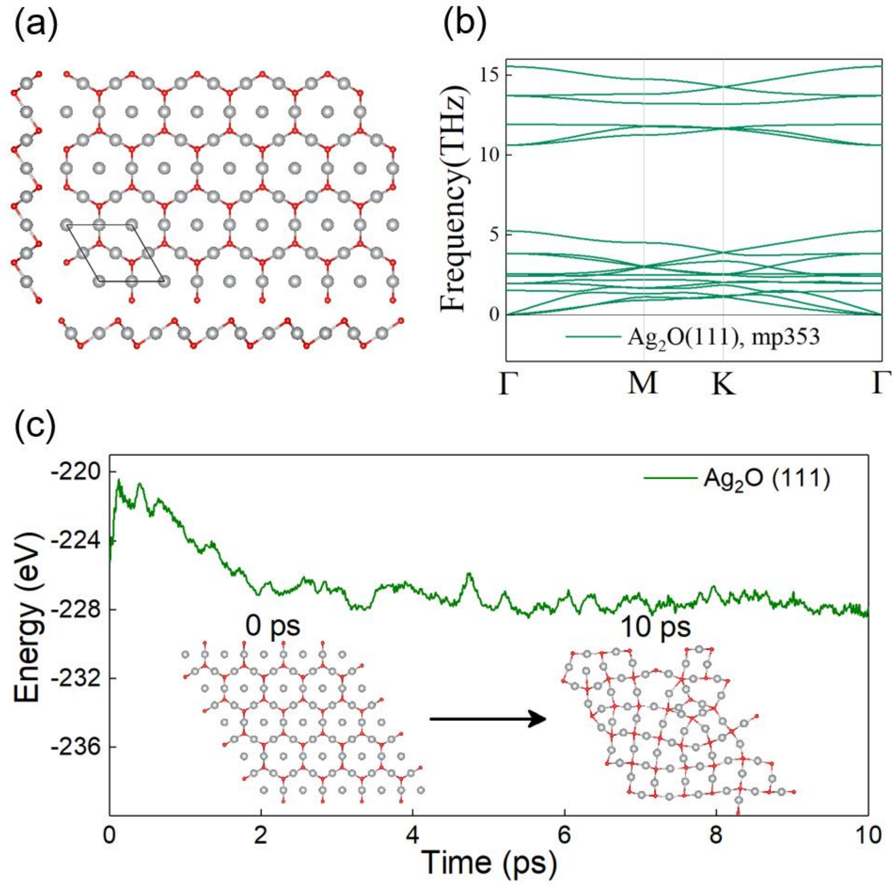

Supplementary Figure 28. **Crystal structure, phonon dispersion and AIMD simulation.** (a) Orthographic projections: views of Ag_2O (111) as seen from the x axis (left), the y axis (bottom) and the z axis (center). The primitive cell is marked by solid box. There are two positions of Ag in Ag_2O (1 1 1): one bonds to oxygen atoms to form a puckered hexagon, and the other exists in isolation at the center of the hexagon. (b) Phonon dispersion: phonon frequencies of Ag_2O (111) along a high-symmetry path. (c) Total system energy fluctuation with simulation time, where insets are crystal structures at 0 ps and 10 ps, respectively.

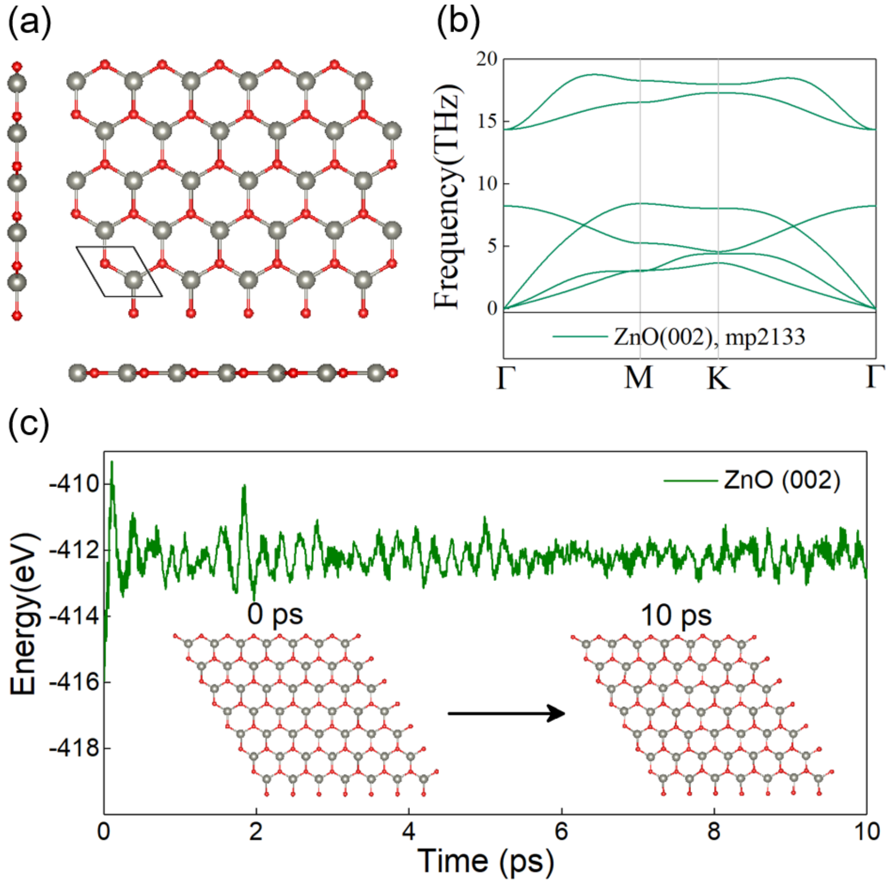

Supplementary Figure 29. Crystal structure, phonon dispersion and AIMD simulation. (a) Orthographic projections: views of ZnO (002) as seen from the x axis (left), the y axis (bottom) and the z axis (center). The primitive cell is marked by solid box. (b) Phonon dispersion: phonon frequencies of ZnO (002) along a high-symmetry path. (c) Total system energy fluctuation with simulation time, where insets are crystal structures at 0 ps and 10 ps, respectively.

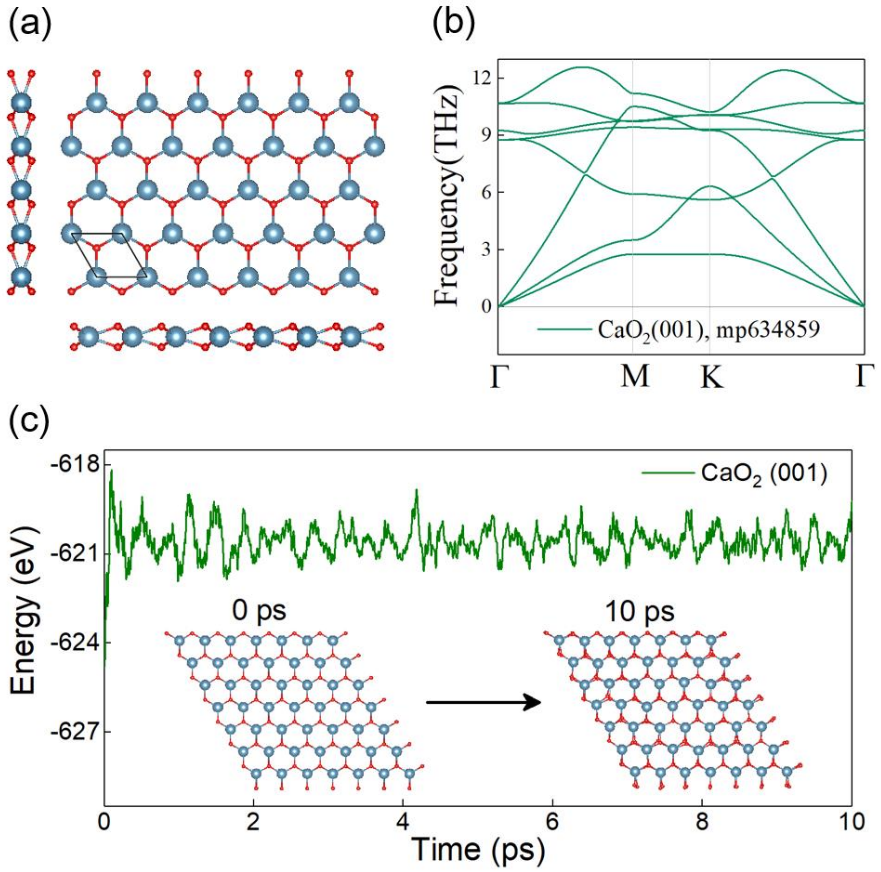

Supplementary Figure 30. Crystal structure, phonon dispersion and AIMD simulation. (a) Orthographic projections: views of CaO_2 (001) as seen from the x axis (left), the y axis (bottom) and the z axis (center). The primitive cell is marked by solid box. (b) Phonon dispersion: phonon frequencies of CaO_2 (001) along a high-symmetry path. (c) Total system energy fluctuation with simulation time, where insets are crystal structures at 0 ps and 10 ps, respectively.

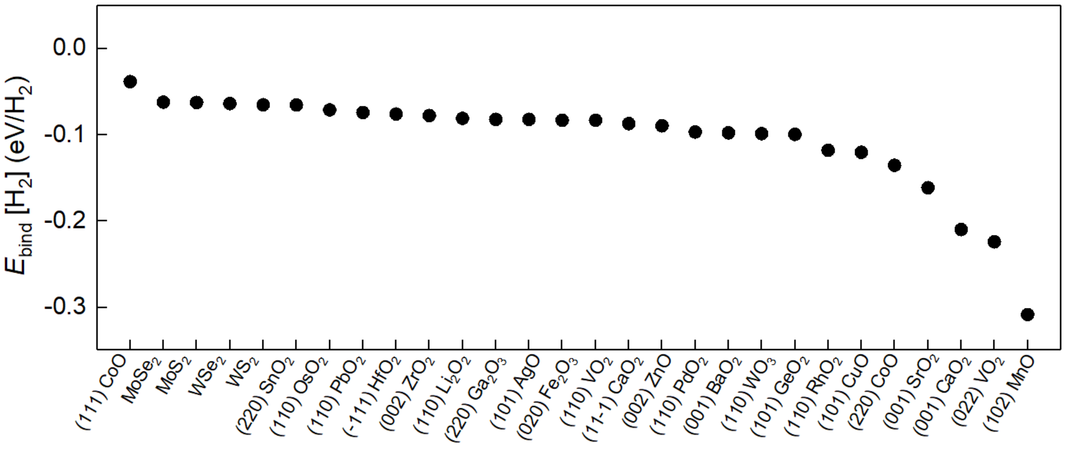

Supplementary Figure 31. Hydrogen binding energy (E_{bind}). Comparison of the typical E_{bind} of the 2D oxides and the other 2D materials TMDs.

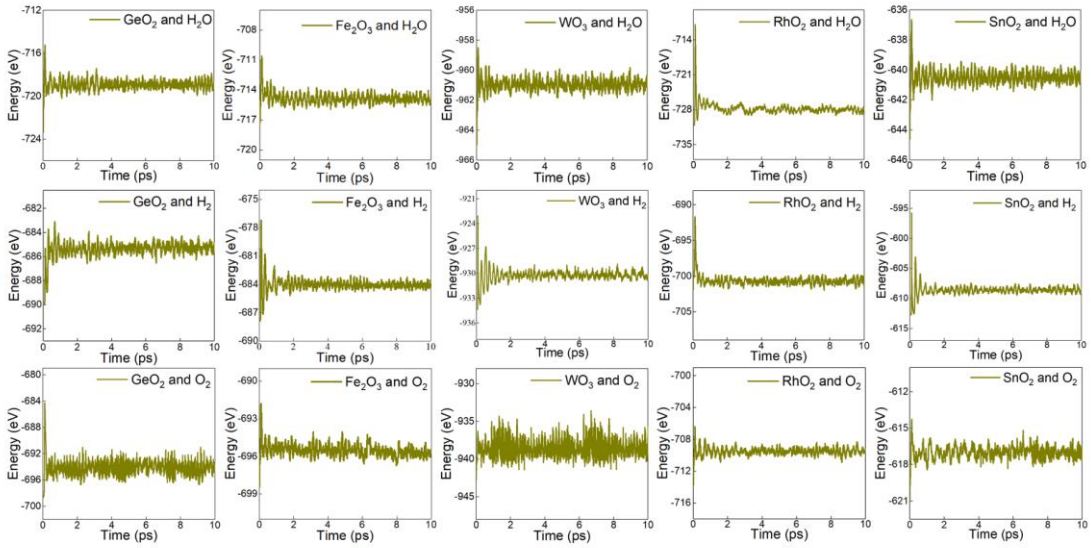

Supplementary Figure 32. Evaluation of surface inertness. AIMD simulation at room temperature for the selected 2D high-κoxides with ambient molecule adsorptions, indicating stable surface without structural collapse.

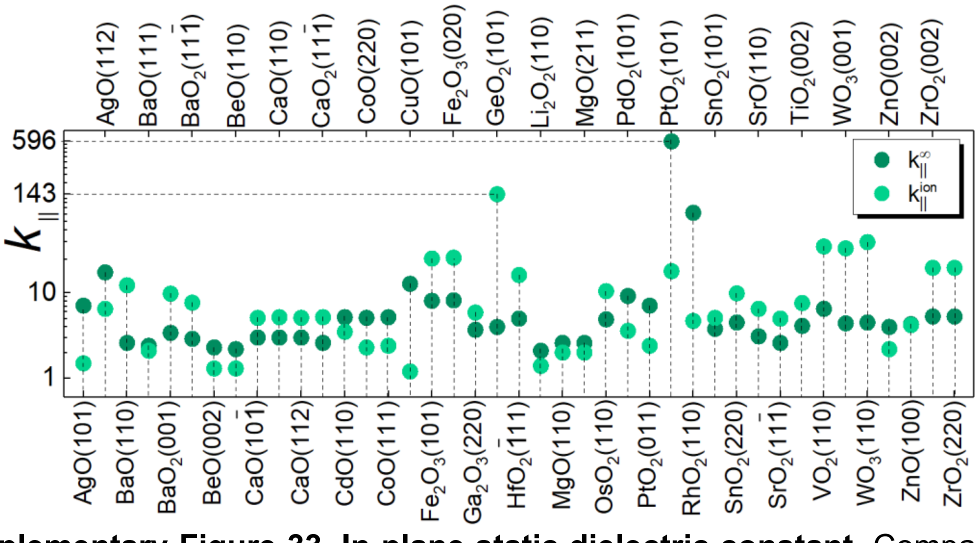

Supplementary Figure 33. In-plane static dielectric constant. Comparison chart for the in-plane electronic and ionic contributions to the dielectric response for 2D semiconductors oxides.

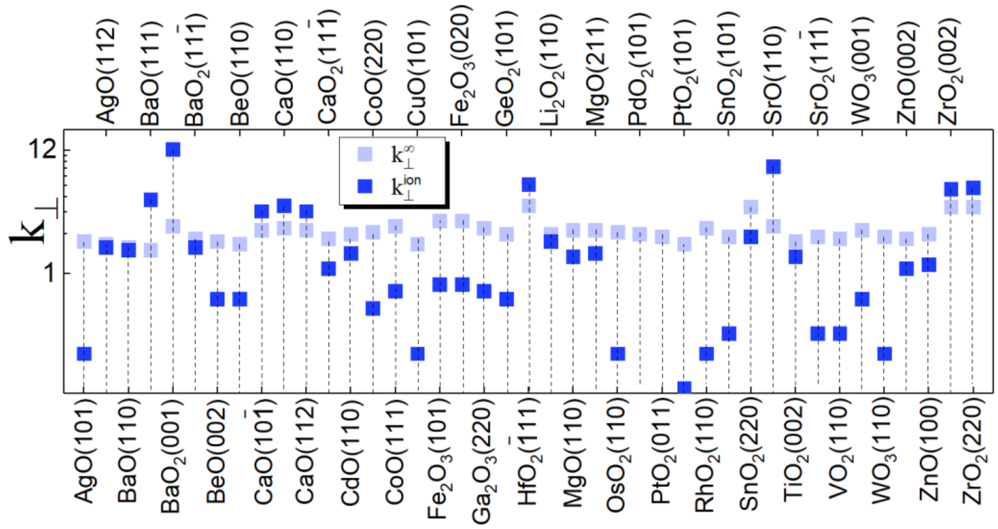

Supplementary Figure 34. Out-of-plane static dielectric constant. Comparison chart for the out-of-plane electronic and ionic contributions to the dielectric response for 47 2D semiconductors oxides.

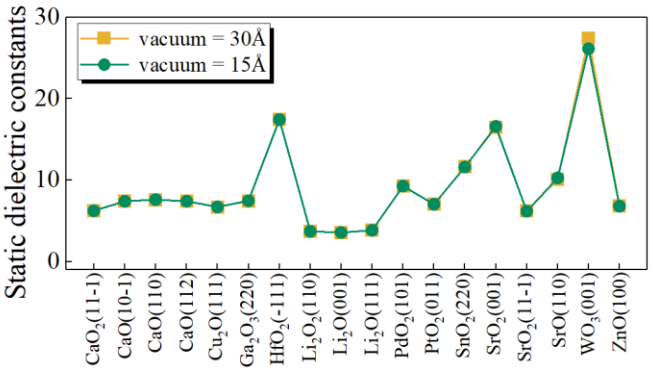

Supplementary Figure 35. Static dielectric constants. Comparison chart for the static dielectric constants with a vacuum size of 15 and 30 Å.

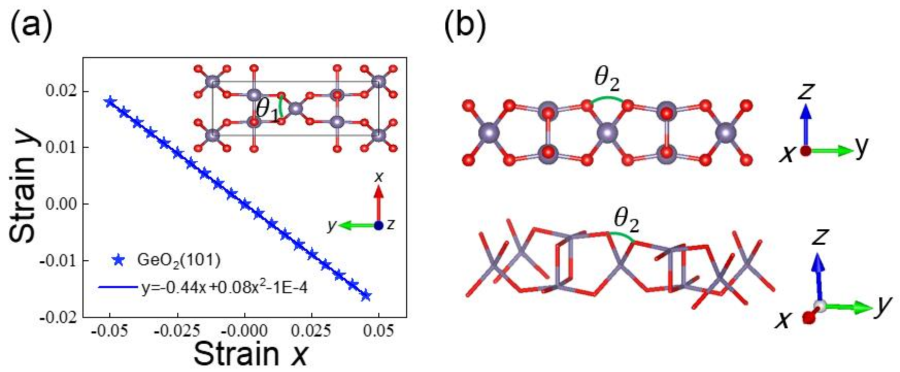

Supplementary Figure 36. Negative Poisson's ratio effect for GeO_2 (101) monolayer. (a) Strain in the *y*-direction induced by the uniaxial strain in the *x*-direction. (b) Crystal structure of GeO_2 monolayer as seen from the *x* axis (top), and the corresponding perspective view (bottom).

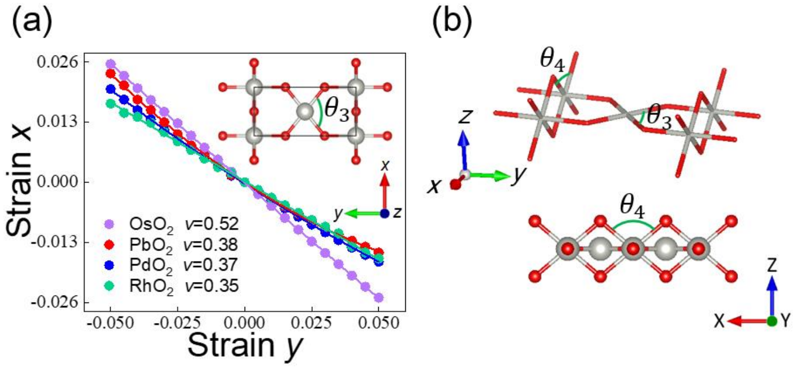

Supplementary Figure 37. Negative Poisson's ratio effect for MO_2 (110) (M=Os, Pb, Pd, Rh) monolayers. (a) Strain in the *x*-direction induced by the uniaxial strain in the *y*-direction. (b) The perspective view of MO_2 (110) monolayer (top), and the corresponding crystal structure as seen from the *y* axis (bottom).

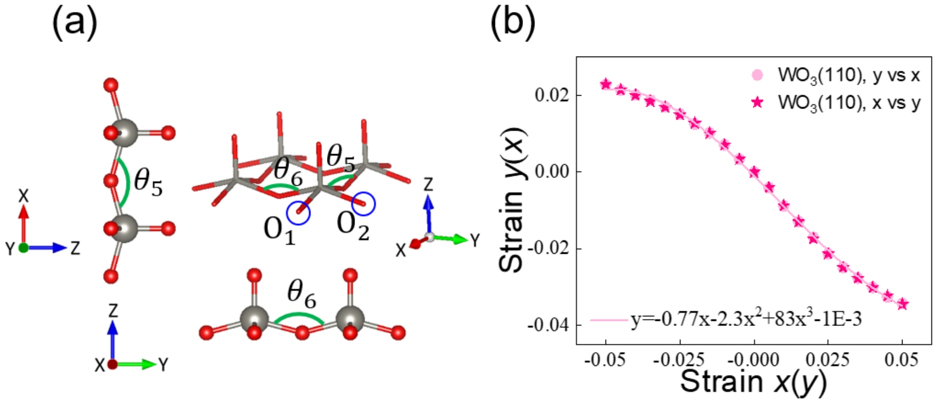

Supplementary Figure 38. Biased Poisson's ratio effect for WO_3 (110) monolayer. (a) Crystal structure of WO_3 (110) monolayer as seen from the *y* axis (left), the perspective (center), and the *x* axis (bottom). (b) Strain in the *y* (*x*) direction induced by the uniaxial strain in the *x* (*y*) direction.

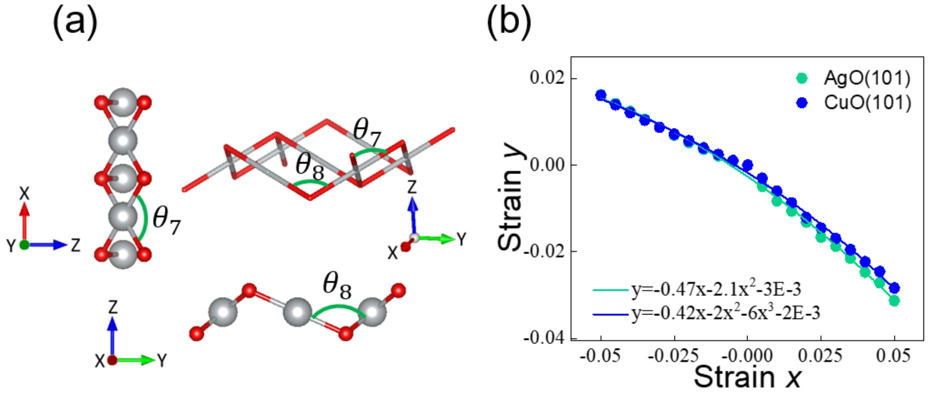

Supplementary Figure 39. Biased Poisson's ratio effect for MO (101) (M=Ag, Cu) monolayers. (a) Crystal structure of MO (101) monolayer as seen from the *y* axis (left), the perspective (center), and the *x* axis (bottom). (b) Strain in the *y*-direction induced by the uniaxial strain in the *x*-direction.

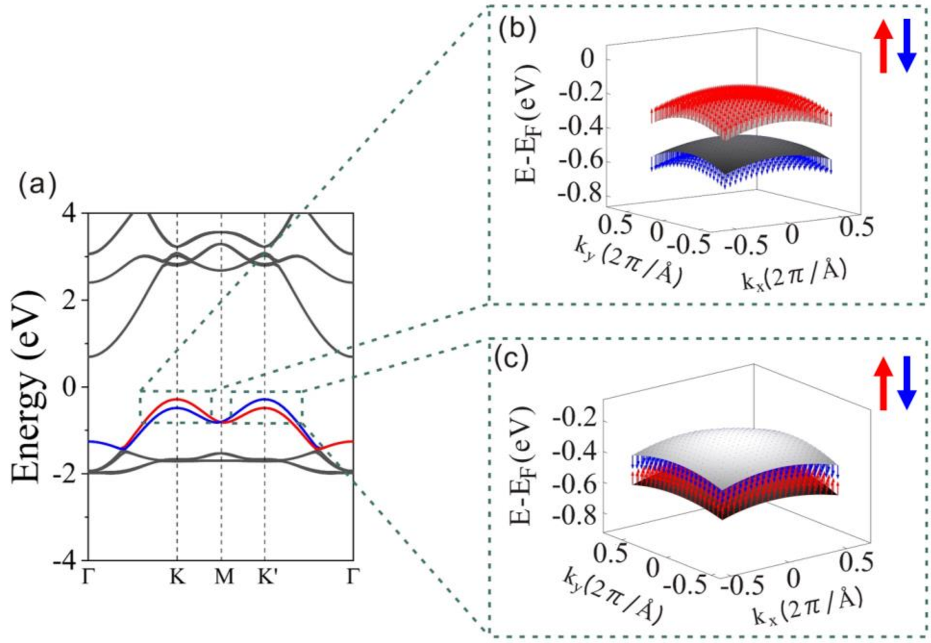

Supplementary Figure 40. Valleytronic Oxide BaO (111). Band structures with spin-orbital coupling calculated at the PBE level for (a) BaO (111) monolayer, and the bands at the K and K' points in the VBM indicated in the solid boxes exhibit significant spin-splitting. The corresponding spin textures are shown in (b, c), where spin polarization is represented by red (up) and blue (down) arrows.

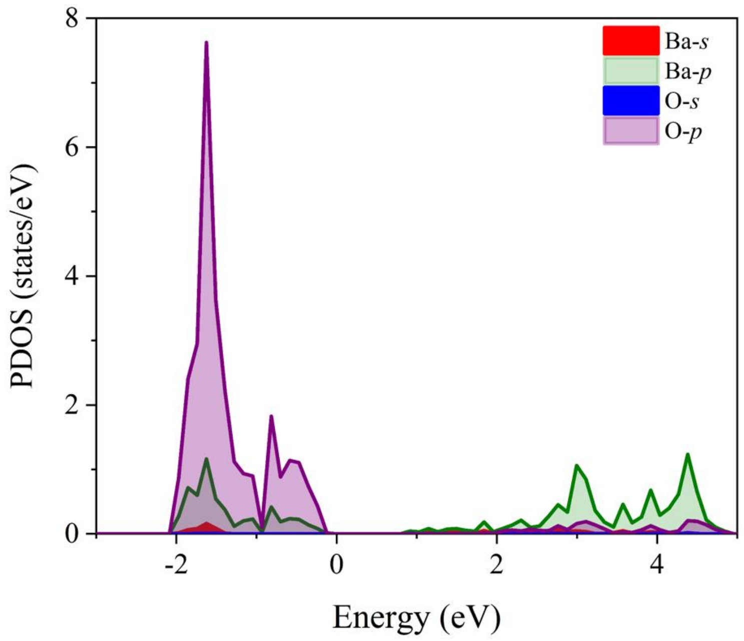

Supplementary Figure 41. Projected density of states (PDOS) for BaO (111). PDOS with spin-orbital coupling calculated at the PBE level.

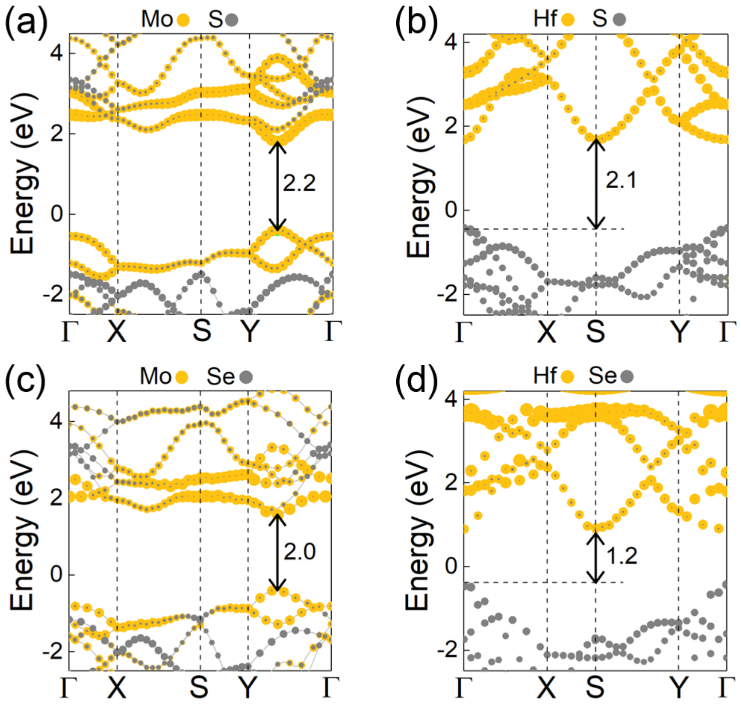

Supplementary Figure 42. Projected band structures calculated with the HSE functional: (a) MoS_2, (b) HfS_2, (c) $MoSe_2$ and (d) $HfSe_2$.

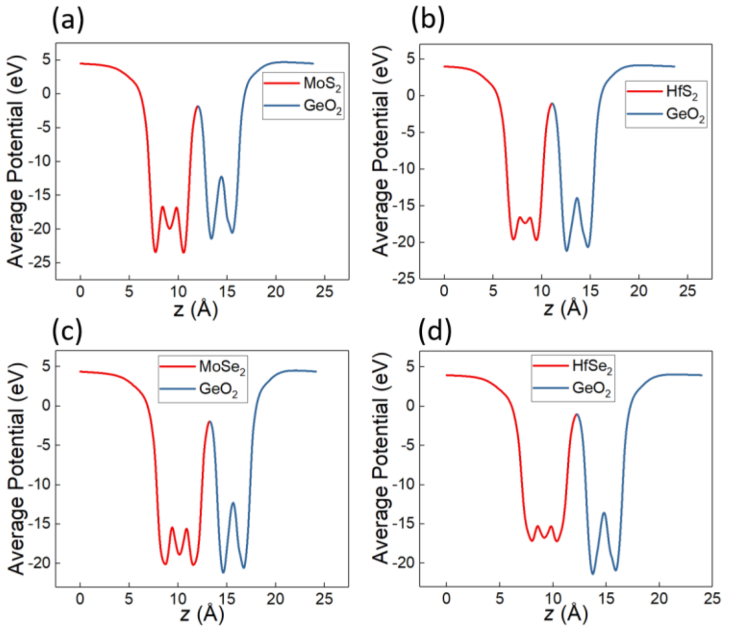

Supplementary Figure 43. Average potential versus *z*-coordinate: (a) GeO_2/MoS_2, (b) GeO_2/HfS_2, (c) $GeO_2/MoSe_2$, and (d) $GeO_2/HfSe_2$ hetero-bilayers.

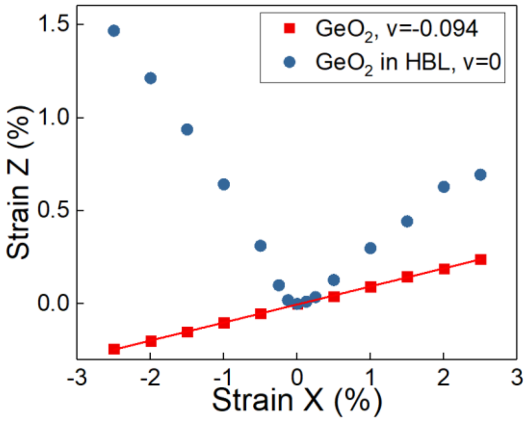

Supplementary Figure 44. Strain curve. Strain Z versus strain X for GeO_2 in $GeO_2/HfSe_2$ HBL and pristine GeO_2 monolayer axis.

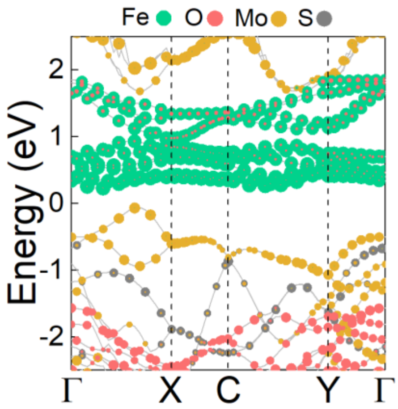

Supplementary Figure 45. Projected band structures of the Fe_2O_3/MoS_2 heterostructure with type-II band alignment. The conduction band minimum (valence band maximum) is located at Fe_2O_3 (MoS_2). A 1×2 supercell of MoS_2 is used to match a 1×1 Fe_2O_3 cell. The strains applied to the Fe_2O_3 along the *x* and *y* directions are 3.5% and 1.9%, respectively.

Supplementary Tables

Supplementary Table 1. U values for d orbitals of transition metal that are fully tested by fitting to experimental lattice constants.

Formula	U	Our work: GGA+U			Experiment			
		a (Å)	b (Å)	c (Å)	a (Å)	b (Å)	c (Å)	ICSD
Ag_2O	5	4.81	4.81	4.81	4.75	4.75	4.75	174090
CoO	3	4.52	4.52	4.52	4.55	4.55	4.55	29082
CrO_2	3	4.42	4.42	2.94	4.42	4.42	2.92	202835
Cu_2O	5	4.29	4.29	4.29	4.22	4.22	4.22	167446
Fe_2O_3	3	5.02	5.02	13.73	5.04	5.04	13.75	173655
HfO_2	3	5.17	5.21	5.35	5.12	5.18	5.30	60903
IrO_2	3	4.54	4.54	3.17	4.50	4.50	3.15	640885
MnO	3	4.43	4.43	4.43	4.44	4.44	4.44	29327
NbO	3	4.22	4.22	4.22	4.21	4.21	4.21	645133

NiO	3	4.15	4.15	4.15	4.17	4.17	4.17	646099
OsO_2	3	4.53	4.53	3.2	4.50	4.50	3.18	30400
PdO	3	3.07	3.07	5.36	3.10	3.10	5.44	185482
PtO_2	3	4.54	4.57	3.15	4.48	4.54	3.14	202407
RhO_2	3	4.46	4.46	3.07	4.49	4.49	3.09	28498
TiO_2	3	12.30	3.76	6.59	12.18	3.74	6.52	41056
VO_2	3	4.45	4.45	2.98	4.53	4.53	2.87	27482
WO_3	3	5.4	5.4	3.93	5.20	5.20	3.84	67748
ZrO_2	3	5.17	5.25	5.36	5.21	5.28	5.39	291451

Supplementary Table 2. Magnetic nonlayered bulk oxides. U values (eV) used for transition metal are given in the second column. The ground-state magnetic configuration (GSMC) is determined by comparing the energy of ferromagnetic (FM) and several antiferromagnetic (AFM) configurations. The corresponding magnetic moment (M) per magnetic atom are given in the last column.

Formula	U	MP ID	GSMC	M (μ_B)
CoO	3	22408	AFM	3.00 (3.35-3.8 ^{Exp})[2,3]
CrO_2	3	19177	FM	2.00 (2 ^{Exp})[4]
CuO	5	1692	AFM	0.98 (0.68 ^{Exp})[3]
Fe_2O_3	3	19770	AFM	5.00 (4.9 ^{Exp})[3]
MnO	3	19006	AFM	5.00 (4.58-4.79 ^{Exp})[3,5]
NiO	3	19009	AFM	2.00 (1.64-1.9 ^{Exp})[3,5]
OsO_2	3	996	AFM	1.95
RhO_2	3	725	FM	0.75
VO_2	3	19094	FM	1.00

Supplementary Table 3. The interplanar spacing (d_{hkl}), close-packed degree and interplanar binding energy (E_b) for 97 crystallographic planes in 47 experimentally stable nonlayered bulk oxides extracted from Materials Project database. The definition of % close-packed can be found from the Methods section in the manuscript.

No.	Formula (*hkl*)	MP ID	d_{hkl} (Å)	% close-packed	E_b (J/m^2)
1	Ag_2O (111)	353	2.8	37.5	1.5
2	Ag_2O (220)	353	1.7	24.9	1.9
3	AgO (002)	499	2.8	31	3.4
4	AgO (101)	499	2.8	37.8	1
5	AgO (112)	499	1.8	27.5	1.3
6	BaO_2 (001)	1105	2.7	13.2	1
7	BaO_2 (11$\bar{1}$)	1105	3.5	13.9	0.7
8	BaO (110)	1342	2.8	100	1.1
9	BaO (111)	1342	3.2	86.6	2.4
10	BeO (002)	2542	2.2	84.5	1.2
11	BeO (110)	2542	1.4	68.8	1.9
12	CaO_2 (001)	634859	2.3	71.6	1.2
13	CaO_2 (11$\bar{1}$)	634859	3.5	69.5	0.5
14	CaO (10$\bar{1}$)	2605	1.7	70.7	2.2
15	CaO (110)	2605	2.4	100	1.8
16	CaO (112)	2605	1.7	70.7	2.2
17	CdO (110)	1132	2.4	100	1.4

18	CoO (002)	22408	2.3	22.9	5.9
19	CoO (111)	22408	2.7	39.1	1.5
20	CoO (220)	22408	1.6	29.8	1.6
21	CrO_2 (011)	19177	3.2	35.8	3.7
22	CrO_2 (200)	19177	1.5	26.3	7
23	Cs_2O_2 (001)	7896	3.7	11.8	0.6
24	Cs_2O_2 $(1\bar{1}1)$	7896	3.3	12.1	0.6
25	CsO_2 (001)	1441	3.2	8.8	0.5
26	CsO_2 $(11\bar{1})$	1441	3.7	8.9	0.4
27	Cu_2O (002)	361	2.1	28	6.1
28	Cu_2O (111)	361	2.5	37.5	1.8
29	CuO (002)	1692	2.6	27.7	7.6
30	CuO (101)	1692	2.5	37.9	2.3
31	Fe_2O_3 (020)	19770	1.9	23	2.4
32	Fe_2O3 (101)	19770	2.7	28.9	2
33	Ga_2O_3 (002)	886	2.9	25.4	6.2
34	Ga_2O_3 (220)	886	3	22	1.3
35	GeO_2 (101)	733	3.5	20.9	2.7
36	HfO_2 (002)	352	2.6	21.6	6.6
37	HfO_2 $(\bar{1}11)$	352	3.2 (3.2^{Exp})[8]	29.3	2.1
38	IrO_2 (101)	2723	2.6	25	4.6
39	IrO_2 (110)	2723	3.2	34.1	3.1
40	K_2O_2 (002)	2672	3.3	12.2	1.1
41	K_2O_2 (112)	2672	2.4	15.3	1.2
42	K_2O (110)	971	3.2	51.3	2.7
43	K_2O (211)	971	2.3	55.6	0.9
44	KO_2 (001)	1866	2.8	15.4	0.7
45	KO_2 $(1\bar{1}0)$	1866	2.8	15.4	0.7
46	KO_2 $(11\bar{1})$	1866	3.4	12.7	0.5
47	Li_2O_2 (101)	841	2.6	45.8	3.7
48	Li_2O_2 (110)	841	1.6	72	2.1
49	Li_2O (001)	1960	2.7	100	2
50	Li_2O (111)	1960	2.7	100	2
51	MgO (110)	1265	2.1	100	2.3
52	MgO (211)	1265	1.5	70.7	2.8
53	MnO (102)	19006	2.2	57.2	1.6
54	Na_2O (211)	2352	2	62.6	1.7
55	Na_2O (220)	2352	1.4	44.3	4
56	NaO_2 (012)	1901	2.4	23.8	2.5
57	NaO_2 (110)	1901	2.7	68.2	0.8
58	NbO (002)	2311	2.1	38.8	4.7
59	NbO (220)	2311	1.5	27.4	8.4
60	NiO (002)	19009	2.4	35.7	5.8
61	NiO (011)	19009	2.1	55.7	2
62	NiO (110)	19009	1.5	39.4	3.3
63	OsO_2 (011)	996	2.6	24.8	3.4
64	OsO_2 (110)	996	3.2	33.5	2.5
65	OsO_2 (200)	996	2.3	19.5	4.2
66	PbO_2 (110)	20725	3.6	34.9	2
67	PbO_2 (200)	20725	2.5	20.6	2.9
68	PdO_2 (002)	1018886	1.6	22.2	2.6
69	PdO_2 (101)	1018886	2.6	23.6	2.6
70	PdO_2 (110)	1018886	3.3	34.9	1.8

71	PdO (002)	1336	2.7	31.7	8.6
72	PdO (011)	1336	2.7	38	2.6
73	PdO (101)	1336	2.7	38	2.6
74	PtO_2 (011)	1285	3.2	35	1.7
75	PtO_2 (101)	1285	2.6	27.8	3
76	Rb_2O_2 (001)	7895	3.5	12.8	0.9
77	Rb_2O_2 ($11\bar{1}$)	7895	3.6	12.1	1.5
78	RbO_2 (001)	12105	3	10.9	0.6
79	RbO_2 ($11\bar{1}$)	12105	3.5	10.4	0.5
80	RhO_2 (101)	725	2.6	23.5	6.3
81	RhO_2 (110)	725	3.2	35	2.9
82	SnO_2 (101)	856	2.7	24	1.5
83	SnO_2 (220)	856	1.7	20.2	2.4
84	SrO_2 (001)	2697	2.5	16.3	1.2
85	SrO_2 ($11\bar{1}$)	2697	3.4	16.6	0.7
86	SrO (001)	2472	3	75.3	6.1
87	SrO (110)	2472	2.6	100	1.5
88	TiO_2 (002)	554278	3.2	22.3	1.4
89	TiO (002)	1071163	1.4	31.7	10.3
90	VO_2 (022)	19094	1.6	23.8	2.4
91	VO_2 (110)	19094	2.5	20.3	2.1
92	WO_3 (001)	19443	3.9	21.3	0.8
93	WO_3 (110)	19443	3.8	21.6	0.9
94	ZnO (002)	2133	2.7	38.9	1.2
95	ZnO (100)	2133	2.8	25.2	2.5
96	ZrO_2 (002)	2858	2.7	19.9	1.7
97	ZrO_2 (220)	2858	1.8	19.8	2

Supplementary Table 4. Magnetic 2D oxides with $E_\mathrm{b} \le 3J/m^2$. Energy difference per formula between antiferromagnetic and ferromagnetic states $\Delta E = E(\mathrm{AFM}) - E(\mathrm{FM})$, positive ΔE value means FM is ground-state magnetic configuration (GSMC), and vice versa. The corresponding magnetic moment (M) per magnetic atom are given in the last column.

System	ΔE (meV)	GSMC	M (μ_B)
CoO (111)	-118.60	AFM	3.00
CoO (220)	-101.40	AFM	3.00
Fe_2O_3 (020)/ (101)	-259.30	AFM	5.00
MnO (102)	-49.00	AFM	4.90
NiO (011)	-103.30	AFM	1.71
OsO_2 (110)	-231.47	AFM	2.85
RhO_2 (110)	-139.70	AFM	1.82
VO_2 (022)	11.10	FM	1.00
VO_2 (110)	5.70	FM	1.00

Supplementary Table 5. 2D oxides in space group $P\bar{1}$ (No. 2, Centrosymmetric (CS)).

General formula	Potentially exfoliable 2D oxides
A_2B_2	Cs_2O_2 (001), K_2O_2 (002), K_2O_2 (112), Rb_2O_2 (001), Rb_2O_2 ($11\bar{1}$)

Supplementary Table 6. 2D oxide in space group Pm (No. 6, non-CS).

General formula	Potentially exfoliable 2D oxides
AB	CoO (220)

Supplementary Table 7. 2D oxides in space group Pc (No. 7, non-CS).

General formula	Potentially exfoliable 2D oxides
AB	VO_2 (022)

Supplementary Table 8. 2D oxides in space group $P2/m$ (No. 10, CS).

General formula	Potentially exfoliable 2D oxides
AB	AgO (101), AgO (112), CuO (101), PdO (011), PdO (101)
AB_2	CsO_2 (001), KO_2 (001), KO_2 ($1\bar{1}0$), PbO_2 (200), RbO_2 (001)

Supplementary Table 9. 2D oxides in space group $C2/m$ (No. 12, CS).

General formula	Potentially exfoliable 2D oxides
AB_2	SrO_2 (001), TiO_2 (002), VO_2 (110)
A_2B_2	Li_2O_2 (110)
A_2B_3	Fe_2O_3 (101), Fe_2O_3 (020)

Supplementary Table 10. 2D oxides in space group $P2_1/c$ (No. 14, CS).

General formula	Potentially exfoliable 2D oxides
AB_2	PtO_2 (101)

Supplementary Table 11. 2D oxides in space group $C222$ (No. 21, Chiral).

General formula	Potentially exfoliable 2D oxides
AB_2	GeO_2 (101)

Supplementary Table 12. 2D oxides in space group $Amm2$ (No. 38, non-CS).

General formula	Potentially exfoliable 2D oxides
AB	CoO (111)

Supplementary Table 13. 2D oxides in space group Pmmm (No. 47, CS).

General formula	Potentially exfoliable 2D oxides
AB_2	OsO_2 (110), PbO_2 (110), PdO_2 (110), RhO_2 (110)
A_2B	K_2O (211), Na_2O (211)
A_2B_2	Cs_2O_2 $(1\bar{1}1)$

Supplementary Table 14. 2D oxides in space group Pmma (No. 51, CS).

General formula	Potentially exfoliable 2D oxides
AB	NiO (011)
A_2B_3	Ga_2O_3 (220)

Supplementary Table 15. 2D oxides in space group Pmmn (No. 59, CS).

General formula	Potentially exfoliable 2D oxides
AB_2	SnO_2 (220)

Supplementary Table 16. 2D oxides in space group P4mm (No. 99, non-CS).

General formula	Potentially exfoliable 2D oxides
AB_3	WO_3 (110)

Supplementary Table 17. 2D oxides in space group P4/mmm (No. 123, CS).

General formula	Potentially exfoliable 2D oxides
AB	BaO (110), CaO $(10\bar{1})$, CaO (110), CaO (112), CdO (110), MgO (110), MgO (211), MnO (102), SrO (110), ZnO (100)
AB_2	BaO_2 (001), BaO_2 $(11\bar{1})$, CaO_2 $(11\bar{1})$, CsO_2 $(11\bar{1})$, KO_2 $(11\bar{1})$, NaO_2 (012), NaO_2 (110), PdO_2 (002), RbO_2 $(11\bar{1})$, SrO_2 $(11\bar{1})$
A_2B	Ag_2O (220), K_2O (110)

Supplementary Table 18. 2D oxides in space group P4/nmm (No. 129, CS).

General formula	Potentially exfoliable 2D oxides
AB_3	WO_3 (001)

Supplementary Table 19. 2D oxides in space group $P\bar{3}m1$ (No. 164, CS).

General formula	Potentially exfoliable 2D oxides
AB_2	HfO_2 ($\bar{1}$11), PdO_2 (101), PtO_2 (011), SnO_2 (101), ZrO_2 (002), ZrO_2 (220)
A_2B	Ag_2O (111), Cu_2O (111), Li_2O (001), Li_2O(111)

Supplementary Table 20. 2D oxides in space group $P\bar{6}m2$ (No. 187, non-CS).

General formula	Potentially exfoliable 2D oxides
AB	BaO (111)[9], BeO (002)[9], BeO (110)[9], ZnO (002)[10,11]
AB_2	CaO_2 (001)

Supplementary Table 21. The comparison between 2D oxides (*h k l*) we predict and those in the C2DB database. Their space group, the root-mean-square deviation (RMSD) of the atomic positions and the difference in total system energy per atom ΔE are given, where $\Delta E = E_{h\,k\,l} - E_{c2db}$. Negative values of ΔE indicate that 2D oxides (*h k l*) are more stable, while positive values indicate the opposite.

Formula	Our work		C2DB Database		RMSD (Å)	ΔE (eV/atom)
	(*h k l*)	No. space	ID	No. space		
AgO	(1 0 1)	10	c2db-832	10	0.0007	Same
	(1 1 2)	10	c2db-832	10	--	0.06
CuO	(1 0 1)	10	c2db-5045	10	0.0085	Same
Ga_2O_3	(2 2 0)	51	c2db-9695	31	--	0.02
GeO_2	(1 0 1)	21	c2db-1454	115	--	0.06
	(1 0 1)	21	c2db-15589	164	--	0.11
HfO_2	($\bar{1}$ 1 1)	164	c2db-4054	115	--	-0.53
	($\bar{1}$ 1 1)	164	c2db-5103	164	0.0026	Same
PbO_2	(1 1 0)	47	c2db-4073	164	--	0.32
	(1 0 1)	164	c2db-4073	164	0.0109	Same
PdO_2	(1 1 0)	47	c2db-14564	164	--	0.31
	(1 0 1)	164	c2db-14564	164	0.0007	Same
	(0 0 2)	123	c2db-14564	164	--	0.61
PtO_2	(0 1 1)	164	c2db-	164	0.0006	Same

			11419			
	(1 0 1)	14	c2db-11419	164	--	0.38
RhO_2	(1 1 0)	47	c2db-5130	115	--	-0.09
SnO_2	(1 0 1)	164	c2db-13529	164	0.0030	Same
	(1 0 1)	164	c2db-12480	115	--	-0.28
	(2 2 0)	59	c2db-13529	164	--	0.09
	(2 2 0)	59	c2db-12480	115	--	-0.22
TiO_2	(0 0 2)	12	c2db-8157	11		-0.28
	(0 0 2)	12	c2db-947	11	--	-0.28
	(0 0 2)	12	c2db-4094	115	--	-0.49
VO_2	(1 1 0)	12	c2db-6195	11	--	-0.22
	(1 1 0)	12	c2db-7244	115	--	-0.31
	(1 1 0)	12	c2db-8293	187	--	-0.45
ZrO_2	(0 0 2)	164	c2db-10398	164	0.0004	Same
	(0 0 2)	164	c2db-9349	115	--	-0.34

Supplementary Table 22. Electronic ground states are characterized with the HSE functional for EE and PE thermodynamic stable 2D oxides (55). Most of 2D oxides (45) are semiconducting, with band gaps ranging from 0.3 to 6.8 eV, including 2 ferromagnetic and 7 antiferromagnetic oxides, while 10 are metallic. Non-centrosymmetric and polar (WO_3 (110)) as well as chiral (GeO_2 (101)) structures are marked by bold font, respectively.

	Semiconductors (41)	Metals (10)
Ferromagnetic (1)	VO_2 (110)	---
Antiferromagnetic (6)	**CoO (111), CoO (220)**, Fe_2O_3 (020), Fe_2O_3 (101), OsO_2 (110), RhO_2 (110)	---
Nonmagnetic (34)	AgO (101), AgO (112), BaO_2 (001), BaO_2 (11$\bar{1}$), BaO (110), **BaO (111), BeO (002), BeO (110)**, CaO_2 (11$\bar{1}$), CaO (10$\bar{1}$), CaO (110), CaO (112), CdO (110), CuO (101), Ga_2O_3 (220), **GeO_2 (101)**, HfO_2 ($\bar{1}$11), Li_2O_2 (110), MgO (110), MgO (211), PdO_2 (101), PtO_2 (011), PtO_2 (101), SnO_2 (101), SnO_2 (220), SrO_2 (11$\bar{1}$), SrO (110), TiO_2 (002), WO_3 (001), **WO_3 (110)**, **ZnO (002)**, ZnO (100), ZrO_2 (002), ZrO_2 (220)	CsO_2 (11$\bar{1}$), KO_2 (11$\bar{1}$), NaO_2 (012), NaO_2 (110), PbO_2 (110), PdO_2 (002), PdO_2 (110), PdO (011), PdO (101), RbO_2 (11$\bar{1}$)

Supplementary Table 23. In-plane (ǁ) and out-of-plane (⊥) static dielectric constants (κ) of 49 2D oxide semiconductors with band gaps ranging from 0.3 to 6.8 eV. κ^{∞} denotes the electronic components of the static dielectric constants (“electronic” + “ionic”). And t is the thickness of monolayer. The thickness t is estimated by the interlayer distance of the bilayer. Band gaps (E_g) are calculated with the HSE functional.

No.	System	t (Å)	$\kappa_{\parallel}^{\infty}$	$\kappa_{\perp}^{\infty}$	$\kappa_{\parallel}$	$\kappa_{\perp}$	E_g (eV)
1	AgO (101)	5.2	7.1	1.9	8.6	2.1	1.3
2	AgO (112)	5.1	17.4	1.8	23.9	3.5	0.7
3	BaO (110)	7.5	2.6	1.7	14.9	3.3	2.1
4	BaO (111)	7.2	2.4	1.6	4.5	6	2.3 (2.2)[9]
5	BaO_2 (001)	4.2	3.4	2.6	13.2	14.7	4.4
6	BaO_2 (11$\bar{1}$)	5.4	2.9	2	10.6	3.7	4.4
7	BeO (002)	4.2	2.3	1.9	3.6	2.5	6.8 (6.8)[9]
8	BeO (110)	4.3	2.2	1.8	3.5	2.4	6.8 (6.8)[9]
9	CaO (10$\bar{1}$)	4.6	3	2.4	8.1	5.9	3.2
10	CaO (110)	4.6	3	2.5	8.2	6.4	3.2
11	CaO (112)	4.6	3	2.4	8.1	5.9	3.2
12	CaO_2 (11$\bar{1}$)	5.2	2.6	2	7.8	3.1	4.9
13	CdO (110)	4.5	5.2	2.2	8.7	3.7	1.1
14	CoO (220)	4.3	5.1	2.3	7.4	2.8	3.7
15	CoO (111)	3.9	5.2	2.6	7.6	3.3	3.8
16	CuO (101)	5.1	12.8	1.8	14.0	2.0	0.6
17	Fe_2O_3 (101)	8	8.1	2.9	33.4	3.7	3.4
18	Fe_2O_3 (020)	8	8.2	2.9	34.1	3.7	3.4
19	Ga_2O_3 (220)	8.1	3.7	2.5	9.6	3.2	4
20	GeO_2 (101)	6.2	4	2.2	147.1	2.8	3.3
21	HfO_2 ($\bar{1}$11)	3.9	5	3.9	21.2	9.9	6.4
22	Li_2O_2 (110)	5.4	2.1	2.2	3.5	4.1	4.5
23	MgO (110)	4.3	2.6	2.4	4.6	3.8	4.3
24	MgO (211)	4.2	2.6	2.4	4.6	3.9	4.3
25	OsO_2 (110)	5.5	4.9	2.3	15.4	2.5	1.7
26	PdO_2 (101)	5.7	9.2	2.2	12.8	2.2	3.1
27	PtO_2 (011)	5.7	7.1	2.1	9.5	2.1	3.3
28	PtO_2 (101)	5.3	596.4	1.8	614.4	1.9	0.5
29	RhO_2 (110)	5.1	86.9	2.5	91.6	2.7	1.4
30	SnO_2 (101)	5.8	3.8	2.1	8.9	2.4	4.1
31	SnO_2 (220)	6.6	4.5	3.8	14.4	5.9	3.7
32	SrO (110)	4.7	3.1	2.6	9.6	11.2	2.8
33	SrO_2 (11$\bar{1}$)	5.3	2.6	1.9	7.6	3.3	4.8
34	TiO_2 (002)	5.6	4.1	2.1	11.7	2.4	4.5
35	VO_2 (110)	5.9	6.5	2	41.5	2.3	1.9
36	WO_3 (001)	6.1	4.4	2.4	37.7	3	1.7
37	WO_3 (110)	5.8	4.5	2.1	43.9	2.3	1.9
38	ZnO (002)	4.4	4	2	6.2	3.1	3.1
39	ZnO (100)	4.2	4.3	2.2	8.5	3.4	2.2
40	ZrO_2 (002)	4	5.3	3.8	25	9.3	5.9
41	ZrO_2 (220)	4	5.3	3.8	25	9.4	5.9

Supplementary Table 24. Total energy per Fe atom for 2D Fe_2O_3 (101) with the magnetic axis oriented in the out-of-plane ($m_\perp$) and in-plane ($m_\parallel$) directions.

Material	E($m_\perp$) meV/Fe-atom	E($m_\parallel$) meV/Fe-atom
Fe_2O_3 (101)	-17196.299	-17196.252

Supplementary Table 25. K-point dependence of maximum and minimum optical phonon modes for Fe_2O_3 (101), OsO_2 (110), and BaO_2 (001) monolayers.

system	Supercell	KP-spacing ($Å^{-1}$)	KP-mesh	Max mode (THz)	Min mode (THz)
BaO_2 (001)	$4 \times 4 \times 1$	0.04	$7 \times 7 \times 1$	24.2406	4.0623
		0.02	$13 \times 13 \times 1$	24.2412	4.0721
		0.01	$27 \times 27 \times 1$	24.2410	4.0722
Fe_2O_3 (101)	$2 \times 3 \times 1$	0.04	$5 \times 8 \times 1$	19.2607	2.6043
		0.02	$9 \times 17 \times 1$	19.2598	2.6035
		0.01	$19 \times 34 \times 1$	19.2599	2.6036
OsO_2 (110)	$2 \times 2 \times 1$	0.04	$4 \times 4 \times 1$	22.4131	2.2383
		0.02	$8 \times 8 \times 1$	22.4132	2.2377
		0.01	$17 \times 15 \times 1$	22.4132	2.2376

Supplementary Table 26. K-point dependence of average dielectric constant for BaO_2 (001), Fe_2O_3 (101), GeO_2 (011), SnO_2 (220), and SrO_2 (001). The standard conventional cell for each material is used for the static dielectric constants calculation.

system	KP-spacing ($Å^{-1}$)	KP-mesh	*k*
BaO_2 (001)	0.02	$13 \times 13 \times 1$	13.70
	0.01	$27 \times 27 \times 1$	13.79
Fe_2O_3 (101)	0.02	$9 \times 17 \times 1$	23.96
	0.01	$19 \times 34 \times 1$	24.00
GeO_2 (101)	0.02	$5 \times 12 \times 1$	98.98
	0.01	$9 \times 25 \times 1$	97.50
SnO_2 (220)	0.02	$12 \times 16 \times 1$	11.61
	0.01	$25 \times 31 \times 1$	11.59
SrO_2 (001)	0.02	$14 \times 13 \times 1$	16.62
	0.01	$28 \times 27 \times 1$	17.21

Supplementary Table 27. Cell lattice type in hexagonal (in-plane lattice parameters: a=b, γ=120°): HfO_2 ($\bar{1}$11) and ZrO_2 (022). Two independent stiffness tensor components C_{11}, C_{12} are given. The elastic constants meet the elastic stability conditions, $C_{11} > 0$ and $C_{11} > |C_{12}|$[12].

2D oxides	C_{11}	C_{12}
HfO_2 ($\bar{1}$11)	160.98	33.57
ZrO_2 (002)	155.68	36.33

Supplementary Table 28. Cell lattice type in rectangular (in-plane lattice parameters: a≠b, γ=90°): Fe_2O_3 (020), GeO_2 (011), OsO_2 (110), PbO_2 (110), PdO_2 (110), RhO_2 (110), AgO (101) and CuO (101). Four independent stiffness tensor components C_{11}, C_{12}, C_{22} and C_{66} are given. The elastic constants meet the elastic stability conditions, $C_{11} > 0$, $C_{66} > 0$ and $C_{11} \cdot C_{22} > C_{12} \cdot C_{12}$ [12].

2D oxides	C_{11}	C_{12}	C_{22}	C_{66}
Fe_2O_3 (020)	139.04	102.53	182.94	45.01
GeO_2 (011)	134.04	50.46	114.45	53.36
OsO_2 (110)	151.84	78.59	176.47	36.2
PbO_2 (110)	100.11	40.26	70.64	9.45
PdO_2 (110)	152.05	56.57	149.08	29.46
RhO_2 (110)	141.28	56.72	159.76	32.7
AgO (101)	58.8	7.37	11.87	3.13
CuO (101)	22.84	8.14	70.94	7.68

Supplementary Table 29. Cell lattice type in square (in-plane lattice parameters: a=b, γ=90°): WO_3 (110). Three independent stiffness tensor components C_{11}, C_{12} and C_{66} are given. The elastic constants meet the elastic stability conditions, $C_{11} > 0$, $C_{66} > 0$ and $C_{11} > | C_{12}|$[12].

2D oxides	C_{11}	C_{12}	C_{66}
WO_3 (110)	91.43	72.36	32.82

Supplementary Table 30. The relaxed lattice parameters for GeO_2 and the rectangular cell of transition metal dichalcogenides.

TMDs	a (Å)	b (Å)	γ (°)
GeO_2	4.01	10.9	90
MoS_2	3.15[13-15]	5.46	90
$MoSe_2$	3.24[13-15]	5.61	90
HfS_2	6.27	3.62	90
$HfSe_2$	6.33	3.65	90

Supplementary Table 31. The interfacial distances for 2D high-*k* GeO_2 (011)/transition metal dichalcogenides heterobilayers.

2D dielectric	2D semiconductor	d_0 (nm)
GeO_2 (011)	MoS_2	0.15
	$MoSe_2$	0.15
	HfS_2	0.17
	$HfSe_2$	0.16

Supplementary Table 32. Band gaps are calculated with the HSE functional for 2D transition metal dichalcogenides, which are in good agreement with the literature values.

Monolayer	Band gap (HSE06)	Band Gap (Refs)
MoS_2	2.2 eV	2.1 eV (HSE06)[16]
$MoSe_2$	2.0 eV	1.6 eV (Exp)[17]
HfS_2	2.1 eV	2.4 eV (HSE06)[18]
$HfSe_2$	1.2 eV	1.3 eV (HSE06)[18]

Supplementary Table 33. The band gaps (E_g) and static dielectric constants (k) of GeO_2 under different strains along the *x*-direction.

Strain X (%)	E_g	k
0	3.29	99
0.5	3.41	151.2
1	3.42	29.9
1.5	3.43	19.4
2	3.42	14.8

Supplementary Table 34. Gibbs free energy (ΔG) of formation for metal oxides. Data from Ref. [8].

Formula	ΔG (kJ/mol)
HfO_2	-1088.2
ZrO_2	-1042.8
Ga_2O_3	-998.3
TiO_2	-888.8
In_2O_3	-830.7
WO_3	-764
Fe_2O_3	-742.2
CaO	-603.3
BeO	-580.1
MgO	-569.3
SrO	-561.9
Li_2O	-561.2
GeO_2	-521.4
BaO	-520.3
SnO_2	-515.8
Bi_2O_3	-493.7

Supplementary References

1. Jain, A. et al. Commentary: The materials project: a materials genome approach to accelerating materials innovation. *APL Mater.* **1**, 011002 (2013).
2. Khan, D. C. & Erickson, R. A. Magnetic form factor of Co^{++} ion in cobaltous oxides. *Phys. Rev. B* **1**, 2243-2249 (1970).
3. Wang, L. et al. Oxidation energies of transition metal oxides within the GGA+U framework. *Phys. Rev. B* **73**, 195107 (2006).
4. Huang, D. et al. Orbital magnetic moments of oxygen and chromium in CrO2. *Phys. Rev. B* **66**, 174440 (2002).
5. Cheetham, A. K. & Hope, D. A. O. Magnetic ordering and exchange effects in the antiferromagnetic solid solutions $Mn_xNi_{1-x}0$. *Phys. Rev. B* **27**, 6964-6967 (1983).
6. Mounet N. et al. Two-dimensional materials from high-throughput computational exfoliation of experimentally known compounds. *Nat. nanotechnol.* **13**, 246-252 (2018).
7. Björkman T. et al. Van der Waals bonding in layered compounds from advanced density-functional first-principles calculations. *Phys. Rev. Lett.*

108, 235502 (2012).
8. Zavabeti A. et al. A liquid metal reaction environment for the room-temperature synthesis of atomically thin metal oxides. *Science* **358**, 332-335 (2017).
9. Luo B.C. et al. Graphene-like monolayer monoxides and monochlorides. *P. Natl. Acad. Sci. USA* 116, 17213-17218 (2019).
10. Hu F.F. et al. ZnO/WSe_2 vdW heterostructure for photocatalytic water splitting. *J. Mater. Chem. C* **7**, 7104-7113 (2019).
11. Tu, Z. C. First-principles study on physical properties of a single ZnO monolayer with graphene-like structure. *J Comput. Theor. Nanos*. **7**, 1182-1186 (2010).
12. Maździarz, M. Comment on 'The computational 2D materials database: high-throughput modeling and discovery of atomically thin crystals'. *2D Mater.* **6**, 048001 (2019).
13. Böker, Th. et al. Band structure of MoS_2, $MoSe_2$, and α-$MoTe_2$: Angle-resolved photoelectron spectroscopy and ab initio calculations. *Phys. Rev. B* **64**, 235305 (2001).
14. Yun, W. et al. Thickness and strain effects on electronic structures of transition metal dichalcogenides: 2H-MX_2 semiconductors (M= Mo, W; X= S, Se, Te). *Phys. Rev. B* **85**, 033305 (2012).
15. Lee, Changhoon. et al. Density functional theory investigation of the electronic structure and thermoelectric properties of layered MoS_2, $MoSe_2$ and their mixed-layer compound. *J. Solid State Chem.* **211**, 113-119 (2014)
16. Hu, W. et al. Effects of interlayer coupling and electric fields on the electronic structures of graphene and MoS_2 heterobilayers. *J. Mater. Chem. C* **4**, 1776 (2016).
17. Tongay, S. et al. Thermally driven crossover from indirect toward direct bandgap in 2D semiconductors: $MoSe_2$ versus MoS_2. *Nano Lett.* **12**, 5576-5580 (2012).
18. Zhao, Q.Y. et al. Elastic, electronic, and dielectric properties of bulk and monolayer ZrS_2, $ZrSe_2$, HfS_2, $HfSe_2$ from van der Waals density-functional theory. *Phys. Status Solidi B* **254**, 1700033 (2017).
